# Proteomic Exploration of Porcine Oocytes During Meiotic Maturation *in vitro* Using an Accurate TMT-Based Quantitative Approach

**DOI:** 10.3389/fvets.2021.792869

**Published:** 2022-02-07

**Authors:** Baoyu Jia, Decai Xiang, Qingyong Shao, Qionghua Hong, Guobo Quan, Guoquan Wu

**Affiliations:** ^1^Key Laboratory of Animal Gene Editing and Animal Cloning in Yunnan Province, College of Veterinary Medicine, Yunnan Agricultural University, Kunming, China; ^2^Yunnan Provincial Genebank of Livestock and Poultry Genetic Resources, Yunnan Provincial Engineering Laboratory of Animal Genetic Resource Conservation and Germplasm Enhancement, Yunnan Animal Science and Veterinary Institute, Kunming, China

**Keywords:** porcine oocytes, *in vitro* maturation, proteome, TMT, PRM

## Abstract

The dynamic changes in protein expression are well known to be required for oocyte meiotic maturation. Although proteomic analysis has been performed in porcine oocytes during *in vitro* maturation, there is still no full data because of the technical limitations at that time. Here, a novel tandem mass tag (TMT)-based quantitative approach was used to compare the proteomic profiles of porcine immature and *in vitro* mature oocytes. The results of our study showed that there were 763 proteins considered with significant difference−450 over-expressed and 313 under-expressed proteins. The GO and KEGG analyses revealed multiple regulatory mechanisms of oocyte nuclear and cytoplasmic maturation such as spindle and chromosome configurations, cytoskeletal reconstruction, epigenetic modifications, energy metabolism, signal transduction and others. In addition, 12 proteins identified with high-confidence peptide and related to oocyte maturation were quantified by a parallel reaction monitoring technique to validate the reliability of TMT results. In conclusion, we provided a detailed proteomics dataset to enrich the understanding of molecular characteristics underlying porcine oocyte maturation *in vitro*.

## Introduction

The domestic pig, as an important livestock species, has been thought to be an ideal large animal model for health and disease research due to its similar organ sizes and physiology to humans (1, 2). It is therefore imperative to generate various types of specially designed pigs for applications in agricultural and biomedical research (3), which is dependent on the constant development of reproductive techniques. Moreover, oocytes occupy a vital position in these technical procedures and directly determine their efficiencies (4). The acquirement of high-quality oocytes through *in vitro* maturation (IVM) technique has become increasingly prominent because the potential of oocytes matured *in vitro* still remain in a compromised state due to incomplete cytoplasmic maturation (5, 6). Currently, numerous studies have been conducted to elucidate the complex regulatory mechanisms underlying oocyte maturation in order to improve the IVM efficiency (7), but the existing mechanisms are not comprehensive enough.

Oocyte maturation is the final step of mammal oogenesis during which meiotic resumption occurs as a progression from initial germinal vesicle (GV) breakdown into metaphase II (MII) arrest (8). This process is accompanied by the deposition of maternal RNAs and proteins regarded as one part of the cytoplasmic maturation for successful oocyte maturation, zygotic genome activation and early embryo development (9, 10). The transcriptome allows an overview of gene expression profile to improve our understanding toward molecular mechanisms of oocyte maturation (11). However, the transcriptional activity is minuscule during oocyte maturation (12), and mRNA and protein levels are rarely correlated well, particularly in oocytes (13). Conversely, a large number of proteins are activated or inactivated by post-translational modifications to employ in oocyte maturation such as nuclear reorganization, cytoskeleton rearrangement, organelle architecture, etc (14). These events are accurately controlled by a network of protein interactions and functions. Therefore, more in-depth proteomic research is of great significance for further elucidating the molecular processes during oocyte maturation.

During the past decade, proteomic strategies have been widely applied to explore the maturation mechanisms of mammalian oocytes including human (15), mouse (16, 17), pig (13, 18–20), cattle (21, 22) and buffalo (23, 24). However for porcine oocytes, all previous studies were conducted by two-dimensional gel electrophoresis (2-DE) to identify the protein composition. But this 2-DE technique remains very difficult to detect low molecular weight and low abundance proteins, with a disadvantage in the accurate quantitation from multiple samples (25). So, in these studies there is a maximum of no more than 1,000 proteins identified in porcine oocytes during IVM. An alternative approach with high-throughput technology is necessary to achieve the comprehensive identification and quantification of proteins for porcine oocytes.

Currently, tandem mass tag (TMT) labeling coupled with liquid chromatography tandem mass spectrometry (LC-MS/MS) is becoming a mainstream proteomic technique attracting tremendous attention in various research fields, due to its advantages of high throughput, sensitivity, accuracy, and stability (26, 27). In addition, a novel targeted method, parallel reaction monitoring (PRM) is applicable to the identification and quantification of low abundant proteins with good selectivity and sensitivity (28), and thus has been used to validate the candidate proteins. In our previously published work, these techniques have already been employed to analyze the proteomic changes of porcine oocytes after vitrification (29). Combining these data, the present study aimed to further acquire the proteomic characteristics of porcine oocytes during meiotic maturation *in vitro*, in order to better understand the potential mechanisms underpinning this process with the protein dimension.

## Materials and Methods

All chemicals and reagents used in this study were purchased from Sigma-Aldrich Chemical Company (Shanghai, China) unless otherwise specified.

### Oocyte Collection and IVM

In this study, medium and procedures for oocyte collection and IVM were as described previously (30). Pre-pubertal ovaries were obtained from a local abattoir and transported to the laboratory in saline at 35–37°C within 2 h. Follicular fluid was aspirated from 3–8 mm antral follicles using a syringe with an 18-gauge needle. Cumulus-oocyte complexes (COCs) in sediments were washed two times in Tyrode's lactate-HEPES-polyvinyl alcohol medium (31), then selected under a stereomicroscope (Olympus, Tokyo, Japan). After washing three times in IVM medium, 50–70 COCs with uniform oocyte cytoplasm and over 3 layers of compact cumulus cells were cultured in each well of a 24-well plate (Costar, Corning, NY, USA) containing 500 μL IVM medium for 42–44 h at 39°C in an atmosphere of 5% CO_2_ with saturated humidity. The composition of IVM medium was a tissue culture medium-199 (ThermoFisher Scientific, Grand Island, NY, USA) supplemented with 3.05 mM D-glucose, 0.57 mM cysteine, 0.91 mM sodium pyruvate, 10% (v/v) porcine follicular fluid, 10 ng/mL epidermal growth factor, 0.5 μg/mL each follicle-stimulating hormone, and luteinizing hormone.

### Protein Extraction, Digestion and TMT Labeling

For collecting GV oocytes, COCs were incubated in 0.1% hyaluronidase for 10 min at 39°C and then mechanically stripped of cumulus cells by repeated aspiration with a 200-μL pipette. In addition, after 42–44 h of IVM, oocytes were also freed from cumulus cells, and those with a first polar body (a characteristic of MII stage) were used for experiments. Both GV and MII oocytes were washed three times in cold Dulbecco's phosphate buffered saline containing 0.3% (w/v) polyvinyl alcohol and stored at −80°C. Three biological replicates were carried out and about 1,500 oocytes were used for each sample.

For protein extraction, these samples were lysed on ice in 8 M urea containing 1% protease inhibitor cocktail through a high intensity ultrasonic processor and then centrifugated at 12,000 g at 4°C for 10 min to obtain the supernatant. Protein concentration was measured by a bicinchoninic acid protein assay kit (Pierce, Rockford, IL, USA) following the manufacturer's instructions. Moreover, the protein solution was reduced with 5 mM dithiothreitol at 56°C for 30 min, alkylated with 11 mM iodoacetamide at room temperature for 15 min, and diluted to ensure the urea concentration was less than 2 M. Trypsin was added at 1:50 mass ratio (trypsin: protein) at 37°C overnight for the first digestion and continuously at 1:100 mass ratio (trypsin: protein) for 4 h to complete a post-digestion. After digestion, peptides were desalted on a Strata X C18 SPE column (Phenomenex, Torrance, CA, USA), vacuum-dried, and reconstituted in 0.5 M TEAB. For TMT labeling, one unit of TMT reagent (Thermo Fisher Scientific, Waltham, MA, USA) was reconstituted in acetonitrile to mix with peptides at room temperature for 2 h. Peptides derived from MII oocyte samples were labeled TMT tags of 126, 127N and 127C, and peptides derived from GV oocyte samples were labeled with TMT tags of 128N, 129N and 129C. Finally, the labeled peptide mixtures were desalted and dried under vacuum centrifugation.

### LC-MS/MS Analysis

Firstly, tryptic peptides were separated at a gradient of 8–32% acetonitrile (pH 9.0) over 60 min into 60 fractions using high pH reverse-phase high performance liquid chromatography with an Agilent 300 Extend C18 column (5 μm particles, 4.6 mm ID, 250 mm length; Agilent, Santa Clara, USA), followed by combining into 9 fractions and vacuum-drying. After dissolving in solvent A (0.1% formic acid), these peptides were directly loaded onto a home-made reversed-phase analytical column (15-cm length, 75 μm i.d.) to elute with gradient solvent B (0.1% formic acid in 98% acetonitrile). The linear gradient settings were as follows: 7–16% over 50 min, 16–30% in 35 min, 30–80% in 2 min and 80% for the last 3 min, which was performed on an EASY-nLC 1,000 ultraperformance liquid chromatography (UPLC) system (Thermo Fisher Scientific, Waltham, MA, USA) at a flow rate of 400 nL/min.

The peptides were subjected to nanospray ionization at 2.0 kV voltage and then detected with tandem mass spectrometry (MS/MS) in Q Exactive^TM^ Plus (Thermo Fisher Scientific, Waltham, MA, USA) coupled online to the UPLC system. Precursor and fragment ion spectra were acquired in the high-resolution Orbitrap with 350–1,550 m/z at a resolution of 60,000 and 100 m/z at a resolution of 30,000, respectively. A data dependent scanning mode was used to acquire a mass fragmentation date. Each full mass spectrometry (MS) scan was followed by 20 MS/MS scans (30.0 s dynamic exclusion) corresponding from the ten most abundant precursor ions of full MS for higher-energy collisional dissociation (HCD) fragmentation with 32% normalized collision energy (NCE). In addition, automatic gain control (AGC) was set at 5E4, and maximum injection time (max IT) was 70 ms.

### Database Processing

The resulting MS/MS spectra were processed using Maxquant search engine (v1.5.2.8) against the Sus scrofa UniProt proteome database (40,708 sequences). Moreover, we added a reverse decoy database to reduce the false positive identification results. Trypsin/P was specified as the cleavage enzyme, allowing up to two missing cleavages. The minimum peptide length was specified as seven amino acids, with a maximum of five modifications per peptide. Precursor mass tolerance was 20 ppm and fragment mass tolerance was 5 ppm. Carbamidomethylation on cysteine was specified as fixed modification and oxidation on methionine and N-terminal acetylation as variable modification. The false discovery rate for each peptide was adjusted to <1%, and minimum score for peptides was set to >40. Student's *t*-test was used to analyze statistical significances between two samples, and *P*-value of < 0.05 and fold change of ≥ 1.20 or ≤ 0.83 were set as the threshold for differentially expressed proteins (DEPs).

### Bioinformatics Analysis

Gene Ontology (GO) annotation proteome was derived from the UniProt-GOA database (http://www.ebi.ac.uk/GOA/) based on biological process, cellular component and molecular function (32). Proteins were further searched with the InterProScan software (http://www.ebi.ac.uk/interpro/) if they were not annotated by the UniProt-GOA database. The online service tool KAAS4 was used to annotate the Kyoto Encyclopedia of Genes and Genomes (KEGG) description (33). Furthermore, enrichment analysis was also carried out by using the Metascape software (http://metascape.org) (34).

### PRM Validation

For PRM analysis, we carried out three biological replicates with at least 1,000 oocytes used for each sample. The tryptic digested peptides were prepared according to the procedures described above. Similarly, peptides were dissolved in solvent A and then eluted with gradient solvent B (6–25% over 40 min, 25–35% in 12 min, climbing to 80% in 4 min, and holding at 80% for the last 4 min), at a flow rate of 500 nL/min. Subsequently, the eluted peptides were subjected to a nanospray ionization source (2.2 kV electrospray voltage) followed by Q Exactive^TM^ Plus coupled online to the UPLC. A data-independent acquisition was conducted on an Orbitrap as follows: full MS scan at a resolution of 70,000 with 350–1,060 m/z (AGC, 3E6; max IT, 50 ms) followed by 20 MS/MS scans at a resolution of 17,500 (AGC, 1E5; max IT, 120 ms; isolation window, 1.6 m/z). In addition, 27% NCE with HCD was used to fragment precursor ions. Acquired PRM data were processed through a Skyline software (version 3.6, MacCoss Lab, University of Washington, USA) (35). The target proteins were quantified according to the fragment ion peak area for confirming the TMT results. We selected 12 proteins according to high-confidence peptide and functional importance, including wee1-like protein kinase 2 (WEE2), kinesin-like protein (KIF20A), ubiquitin conjugating enzyme E2 C (UBE2C), DNA (cytosine-5)-methyltransferase (DNMT1), proliferating cell nuclear antigen (PCNA), CD59 glycoprotein (CD59), growth differentiation factor 9 (GDF9), coronin (CORO1C), tudor and KH domain-containing protein (TDRKH), tropomyosin alpha-4 chain (TPM4), annexin (ANXA1) and cytoplasmic polyadenylation element-binding protein 1 (CPEB1).

## Results

### Protein Identification

**Figure 1:**
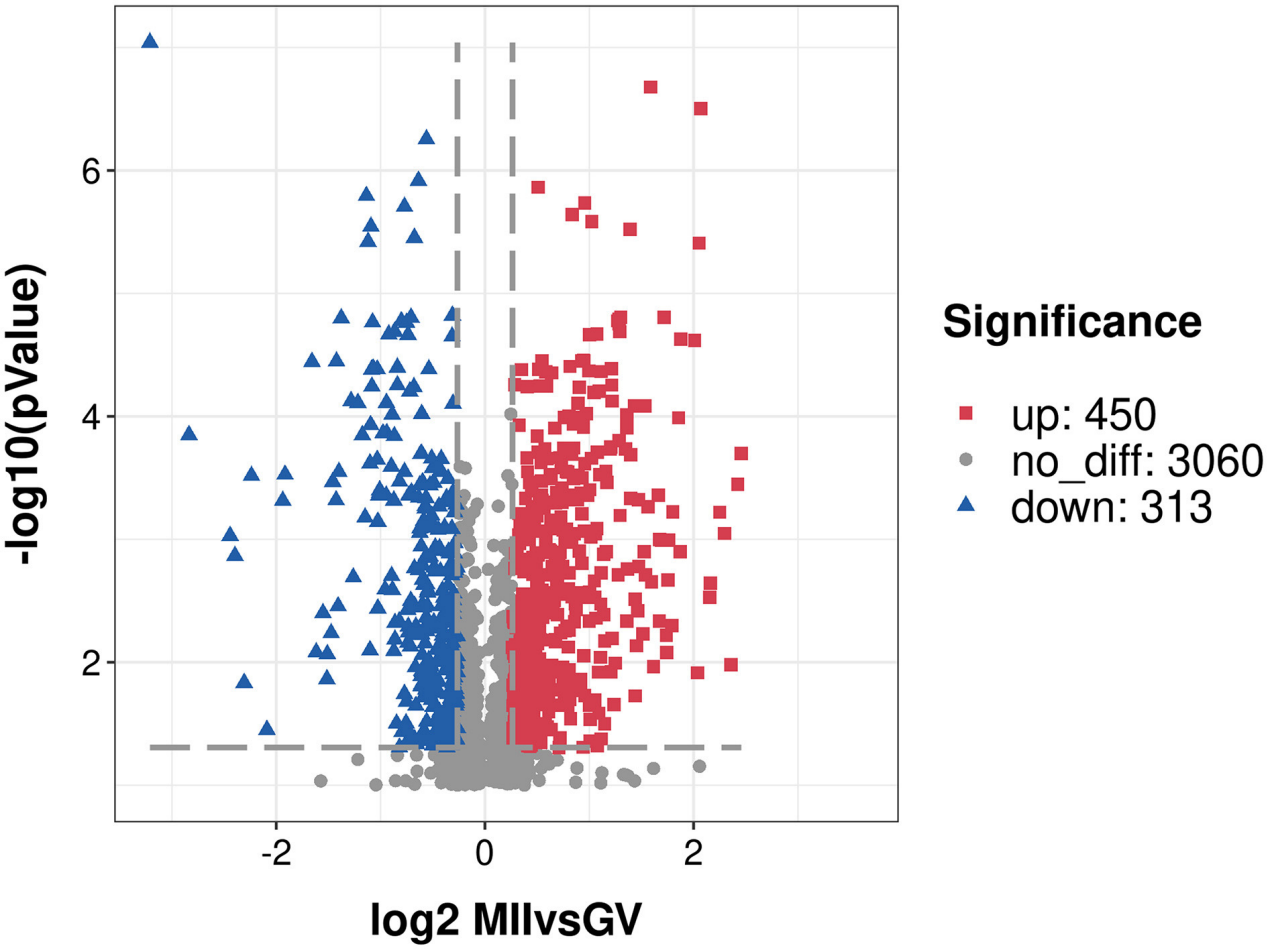
A volcano plot of differentially expressed proteins. Proteins with fold change of ≥ 1.20 or ≤ 0.83 and *P* < 0.05 were considered statistically significant. Red blocks indicate significant over-expressed proteins, blue triangles indicate significant under-expressed proteins, while gray circles indicate proteins without differences. The X-axis represents fold change, Y-axis means *P*-value.

After a stringent criteria for quality control (TMT labeling efficiency of 98.42%, peptide mass error within 5 ppm), we obtained a total of 3,823 proteins with quantitative information and the detailed description is provided in Supplementary Table S1. Moreover, a principal component analysis of all quantified proteins showed two completely independent clusters, indicating that the replicates from each treatment were very close to each other (Supplementary Figure S1). Among these proteins, 763 proteins (*P*-value < 0.05, fold change of ≥ 1.20 or ≤ 0.83) were considered as the DEPs, and 450 proteins were over-expressed, and 313 proteins were under-expressed in the MII oocyte (Supplementary Table S2). A volcano plot indicated the repartition of each protein abundance (Figure 1). In addition, there were 144 DEPs with fold change more than two, including 101 over-expressed and 43 under-expressed proteins.

### Functional Classification Analysis

**Figure 2:**
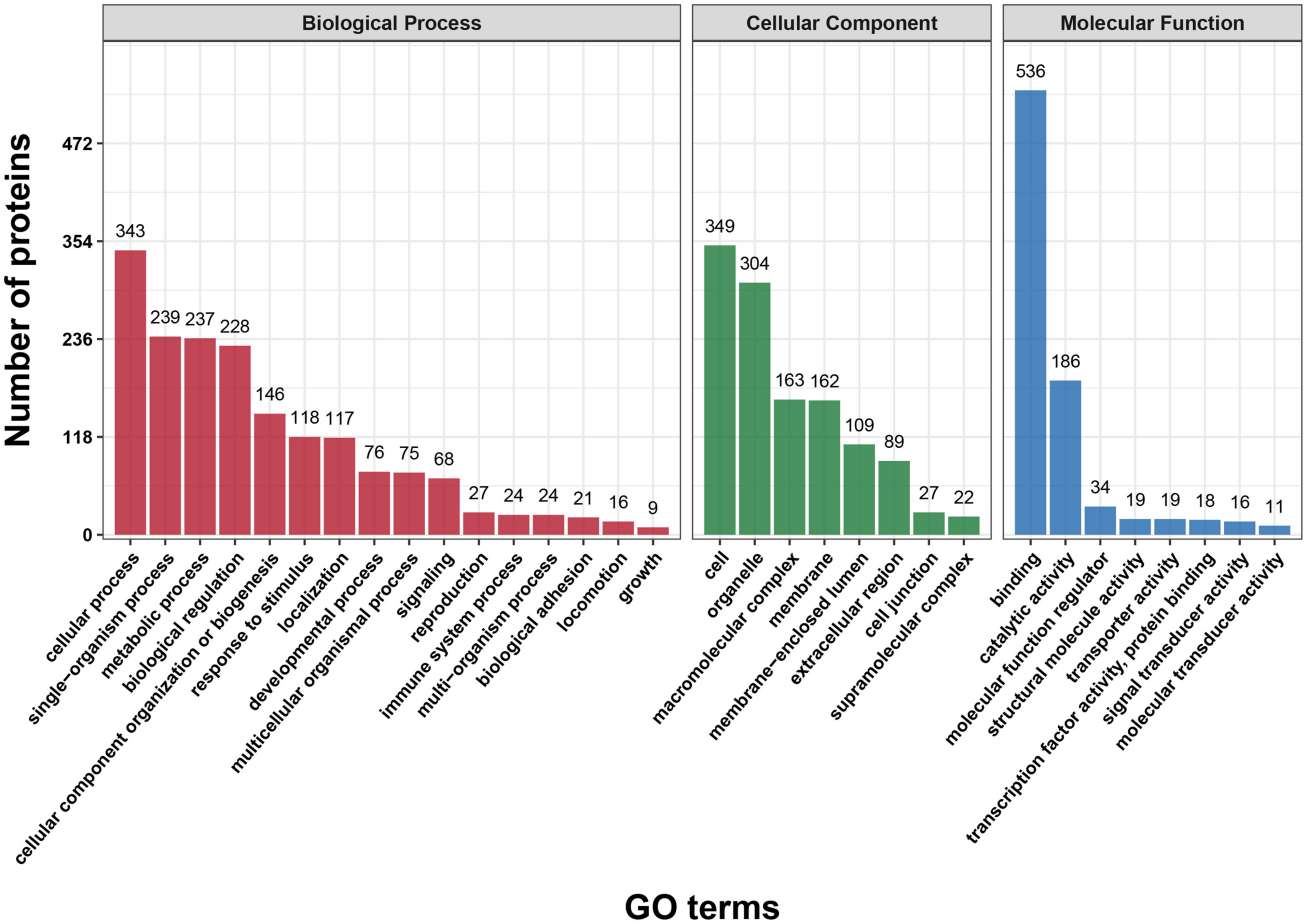
Gene Ontology (GO) classification analysis of differentially expressed proteins (DEPs) in terms of biological process, cellular component and molecular function. Each bar represents the number of DEPs.

First, we performed a GO functional classification on the DEPs and showed 32 terms including 16 biological processes, 8 cellular components and 8 molecular functions (Figure 2 and Supplementary Table S3). For the biological process, the top five GO terms consisted of “cellular process” (343 proteins), “single-organism process” (239 proteins), “metabolic process” (237 proteins), “biological regulation” (228 proteins) and “cellular component organization” (146 proteins). In the cellular component, the most abundant terms were “cell” and “organelle” with 349 and 304 proteins, respectively. The “binding” (536 proteins) and “catalytic activity” (186 proteins) were the largest two terms for molecular function.

**Figure 3:**
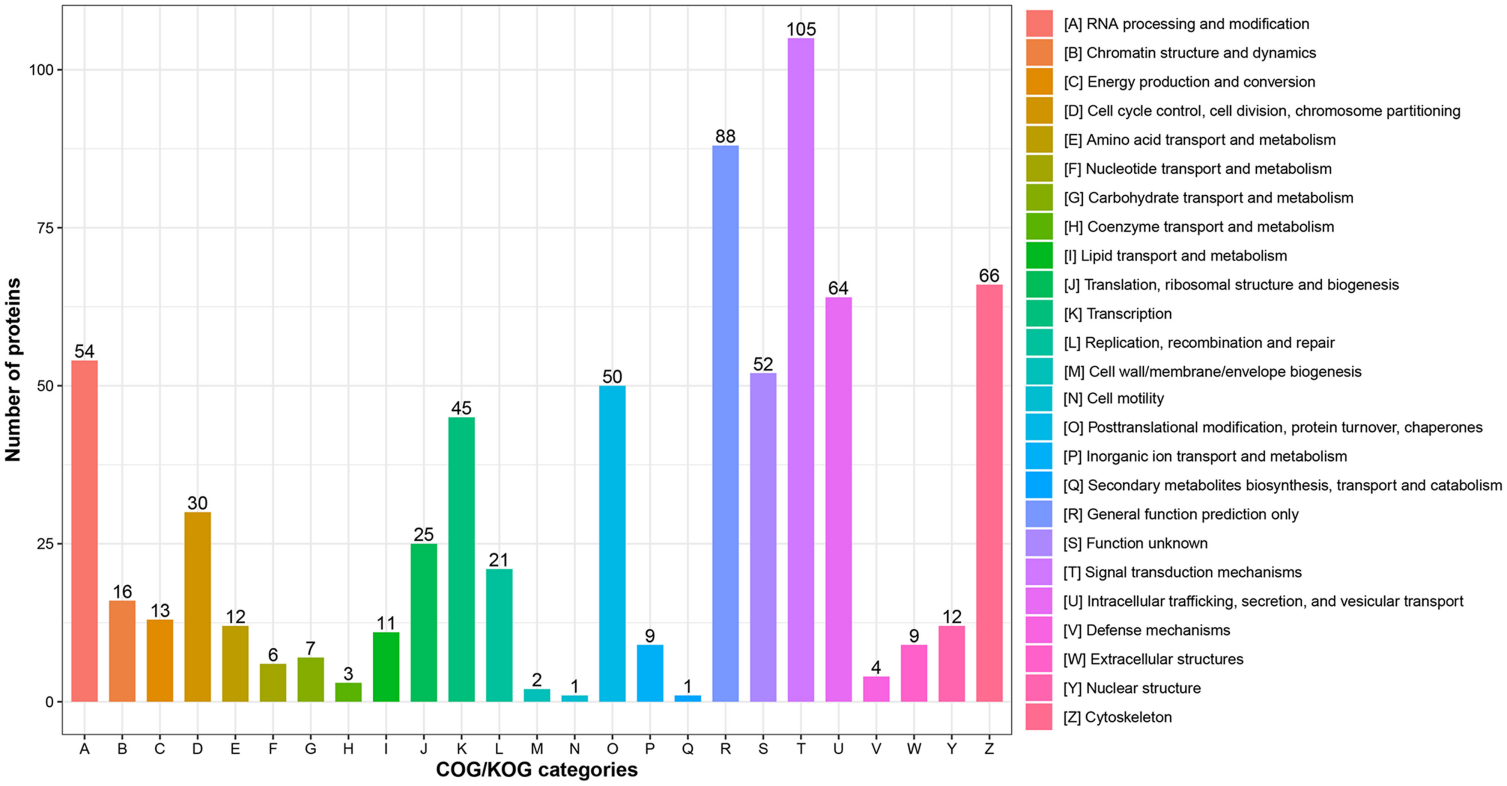
COG/KOG functional classification analysis of differentially expressed proteins (DEPs). The DEPs were aligned to COG/KOG database and classified into 20 functional clusters. Each bar represents the number of DEPs.

In addition, functional classification based on the COG/KOG database was also used to analyze the protein function and characteristic. These DEPs were assigned to 25 COG/KOG categories (Figure 3 and Supplementary Table S4). The largest category was “signal transduction mechanisms” (105 proteins), followed by “general function prediction only” (88 proteins) and “cytoskeleton” (66 proteins). Moreover, some DEPs were clustered in important categories related to oocyte meiosis such as “cell cycle control, cell division, chromosome partitioning” (30 proteins), “chromatin structure and dynamics” (16 proteins) and “nuclear structure” (12 proteins).

### Functional Enrichment Analysis

**Figure 4:**
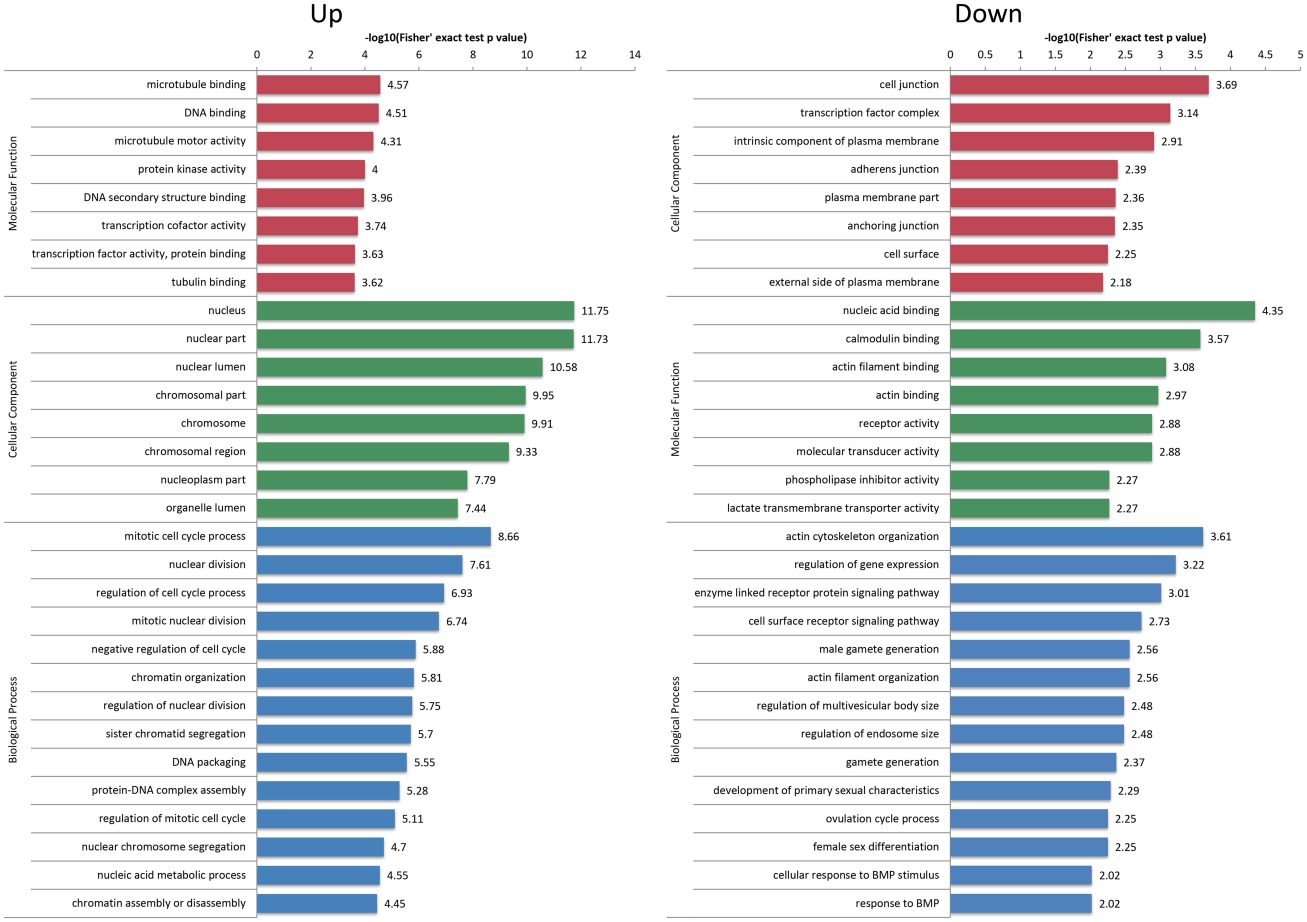
Gene Ontology (GO) enrichment analysis of differentially expressed proteins (DEPs). The over-expressed and under-expressed DEPs were enriched into cellular component, molecular function and biological process. The bar represents the enrichment score associated with each term, and score value is shown as -log10 (Fisher's exact test *P*-value).

First of all, the detailed information of GO functional enrichment is shown in Figure 4 and Supplementary Table S5. Within the molecular function, a number of over-expressed DEPs were enriched for microtubule-related terms including “microtubule binding,” “microtubule motor activity” and “tubulin binding,” whereas “actin filament binding” and “actin binding” terms were under-expressed. A molecular function analysis showed that most of the over-expressed DEPs were involved in the regulation of the nucleus and chromosome, and the under-expressed terms were concerned with “cell junction,” “adherens junction” and “anchoring junction.” Regarding the biological process from over-expressed DEPs, almost all terms related to the cell cycle networks were “mitotic cycle process,” “nuclear division,” “regulation of cell cycle process,” “mitotic nuclear division,” “chromatin organization,” etc. Still there were some under-expressed DEPs associated with “actin cytoskeleton organization” and “actin filament organization” terms.

**Figure 5:**
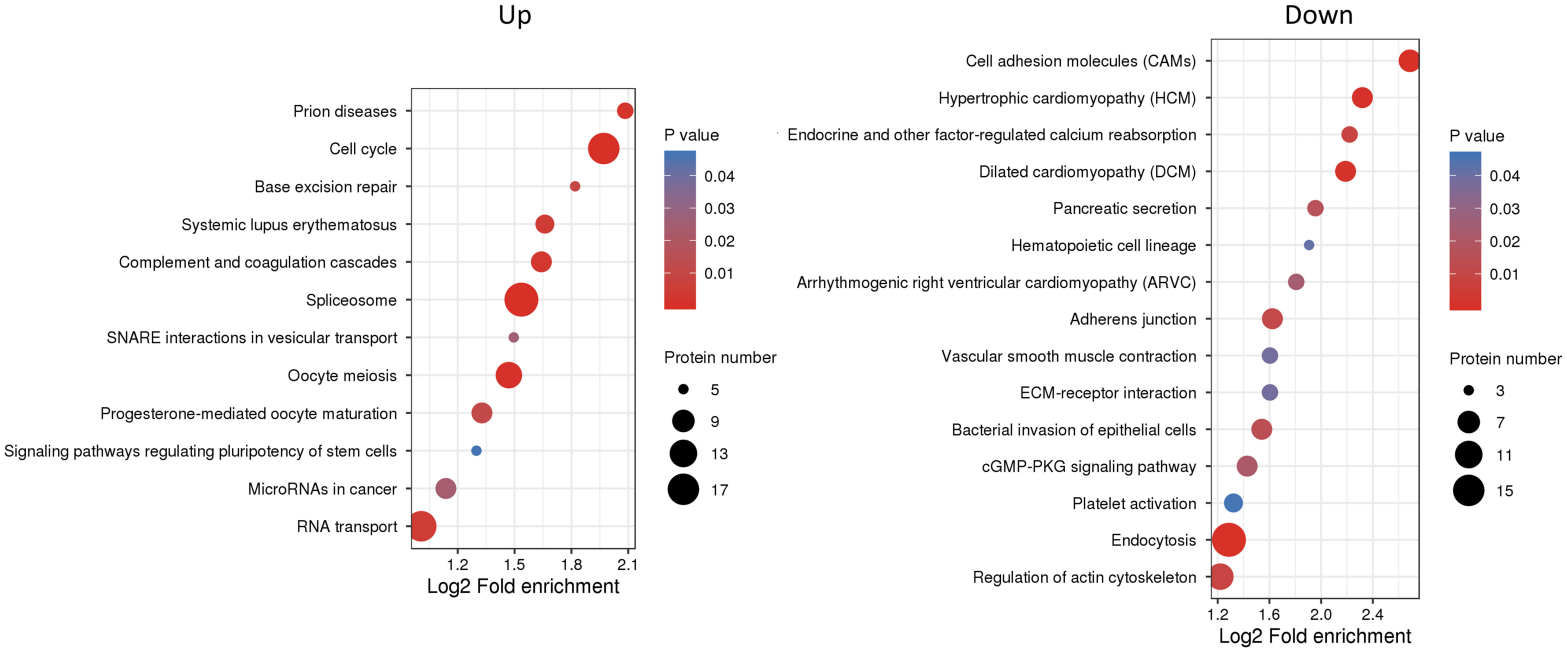
Kyoto Encyclopedia of Genes and Genomes (KEGG) pathway enrichment analysis of differentially expressed proteins (DEPs). The *P*-value was calculated using a Fisher's exact test. The X-axis represents the log2 fold enrichment, Y-axis means KEGG pathway. Bubble size corresponds to the number of DEPs for each term.

We next used KEGG pathway analysis to delineate the protein functions (Figure 5 and Supplementary Table S6). Several signaling pathways related to oocyte maturation were enriched including “oocyte meiosis,” “progesterone-mediated oocyte maturation,” “thyroid hormone signaling pathway,” “insulin signaling pathway,” “MAPK signaling pathway,” etc. Moreover, the under-expressed DEPs enriched the many significant pathways associated with “cell adhesion molecules,” “adherens junction,” “tight junction” and “regulation of actin cytoskeleton.”

**Figure 6:**
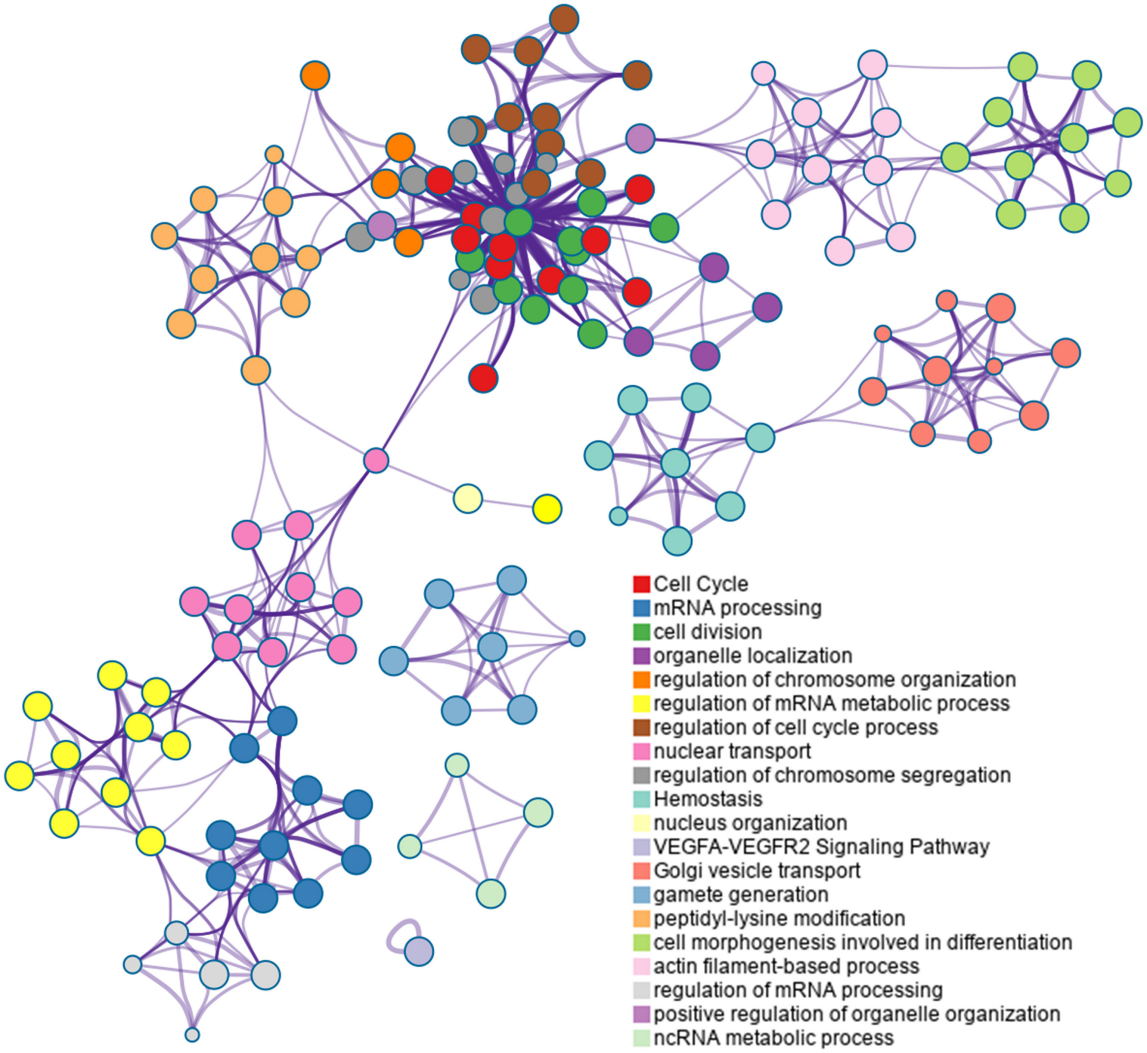
Top 20 clusters in the enrichment network of differentially expressed proteins (DEPs). Metascape is used for annotation and enrichment visualization of DEPs. Each circle represents an enriched term and is colored according to cluster identity. Terms with a similarity score >0.3 are linked by edges.

Based on the Metascape software, the enriched clusters for DEPs included those of “cell cycle,” “mRNA processing,” “cell division,” “organelle localization,” “actin filament-based process” and others (Figure 6 and Supplementary Table S7).

### PRM Validation

**Figure 7:**
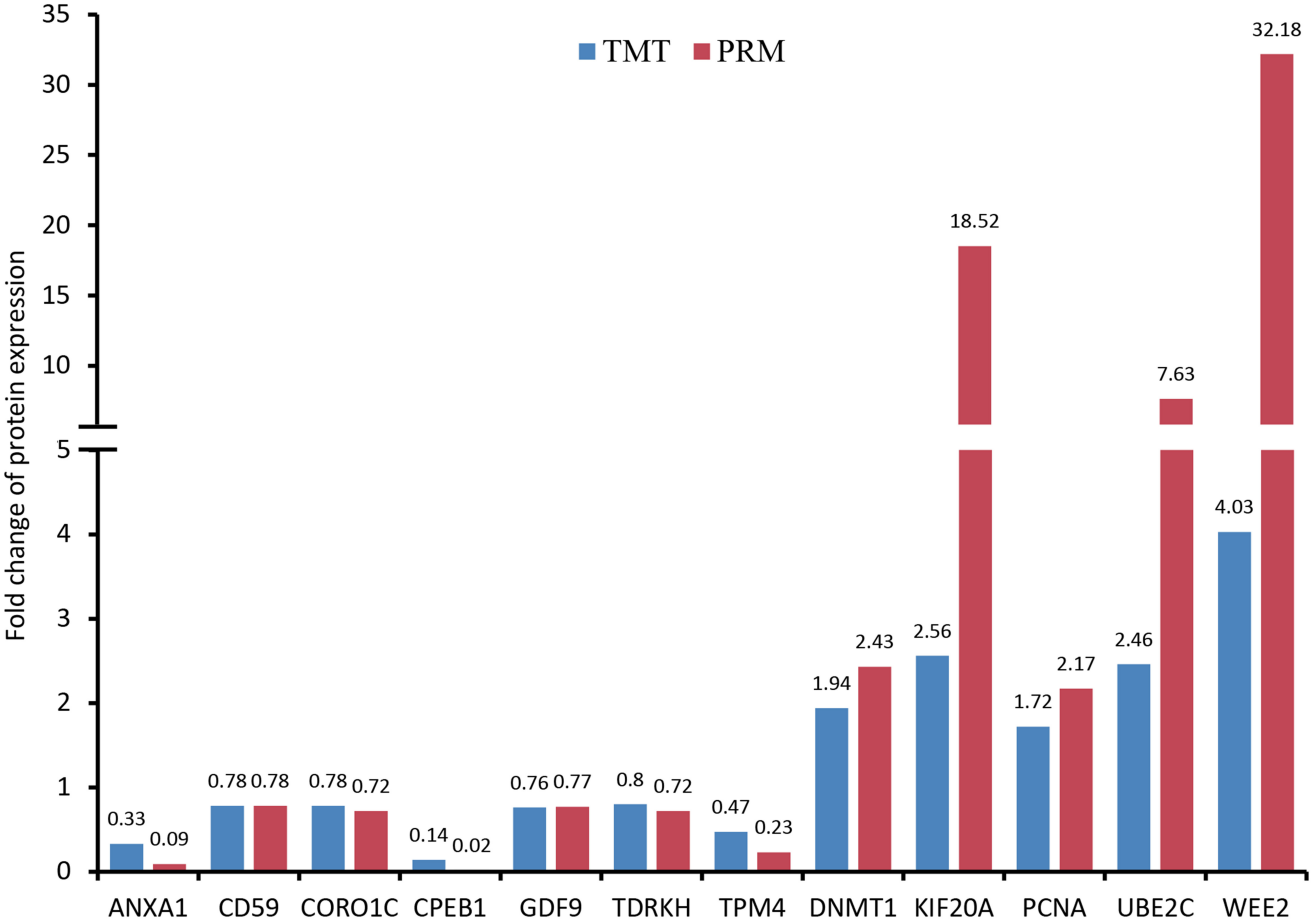
Validation of differentially expressed proteins (DEPs) using parallel reaction monitoring (PRM) analysis. There are 12 candidate DEPs obtained to verify the tandem mass tag (TMT) results.

As shown in Figure 7 and Supplementary Figure S2, the expression patterns of these proteins quantified by PRM and TMT were completely consistent, although the fold change in protein expression levels varied between the two techniques.

## Discussion

There is a large pool of protein composition stored in GV oocytes, which exhibits dynamic changes during the process of meiotic resumption. Accumulation of maternally derived proteins in cytoplasm is critical to oocyte maturation, fertilization and embryo development (36). Thus, an investigation of oocyte proteome variations during the GV to MII transition is of great significance to uncover regulatory proteins and related functional phenotypes of oocyte maturation. The present results showed a series of differential proteins between GV and MII oocytes. A substantial number of known proteins associated with both nuclear and cytoplasmic maturation were further confirmed by the current study. We also found that some novel proteins were supposed to take part in oocyte maturation. Furthermore, bioinformatics interrogation revealed important changes in protein regulation of multiple cellular functions in porcine oocytes during IVM.

Regarding the porcine oocytes, high-throughput technology has not been used for proteome analysis. Our study obtained 763 quantified proteins considered as significant difference in the MII oocytes through TMT-based proteomic approach, which is far more than date of Kim et al. who have reported 16 over-expressed and 12 under-expressed proteins using 2-DE analysis (13). Another study also found only 16 proteins that were differentially expressed during IVM of porcine oocytes (19). Moreover, almost all of these DEPs identified in previously reported studies were not detected in the current study.

Cellular metabolism is vital for oocyte maturation because the large-scale reorganizations of nucleus and cytoplasm require a massive amount of energy from various substrates such as carbohydrates, amino acids, and lipids (37). Currently, both glucose and pyruvate are commonly added to IVM medium to support porcine oocyte maturation and subsequent early embryo development (38). In the present study, there were 237 DEPs classified in the “metabolic process,” some of which were assigned to “energy production and conversion,” “carbohydrate transport and metabolism,” “amino acid transport and metabolism,” “lipid transport and metabolism” and so on, according to COG/KOG categories. These proteins play a crucial role in the regulation of energy metabolism during oocyte maturation. For instance, monocarboxylate transporter 1 (SLC16A1) and solute carrier family 16 member 3 (SLC16A3), belonging to a family of proton-linked monocarboxylate transporters, are involved in the movement of lactate, pyruvate, acetate and ketone bodies, and their regulation and function have been confirmed in preimplantation mouse embryos (39). Thioredoxin domain containing 9 (TXNDC9), as a prominent member of thioredoxins, can maintain the redox state and regulate the folding of actin and tubulin (40). It has been reported that TXNDC9 is required for mouse oocyte maturation and shows a higher protein expression in GV stage compared to MII stage (41). However, we observed the highest level of TXNDC9 protein in porcine oocytes after IVM. Conversely, there are a large number of lipid droplets in porcine oocyte cytoplasm, which have been demonstrated to play a fundamental role in the oocyte maturation process by providing an energy source from lipid metabolism (42, 43). Based on our results, many proteins were uncovered to participate in the signaling pathway of lipid metabolism during oocyte maturation such as methylsterol monooxygenase 1 (MSMO1), phospholipase A2 activating protein (PLAA), phosphatidate phosphatase (LPIN2), acetyl-CoA carboxylase 1 (ACACA), etc. LPIN2 promotes the accumulation of lipid droplets and its degradation is essential for lipolysis activation (44). The under-expressed expression of LPIN2 protein in MII oocytes may also suggest a marked increase in lipid metabolism during the IVM process.

In the biological process, the over-expressed proteins were mainly enriched in the GO terms associated with cell cycle regulation, indicating that they might play a key role in the meiotic progression of oocytes. The KEGG enrichment analysis also identified two pathways, “cell cycle” and “oocyte meiosis,” as significantly enriched. As expected, the present study found some known proteins including CPEB1, KIF20A, kinetochore complex component (NDC80), mitotic checkpoint protein BUB3 (BUB3), UBE2C and others. Previous studies have showed that these proteins mentioned above are essential for meiotic resumption of porcine oocytes such as regulating spindle formation and chromosome alignment (45–49). Incidentally, we observed that securing (PTTG1) expression was over-expressed 4.1-fold in MII oocytes, which may participate in the processes of oocyte maturation and zygotic genome activation as a maternal protein (50). Conversely, several proteins that have not yet been studied in porcine oocytes were also identified to be involved in the cell cycle. For example, WEE2 protein is an oocyte specific tyrosine kinase and is critical for exit from MII arrest and promotes pronuclear formation (51). Structural maintenance of chromosomes protein (SMC1B), a meiosis specific component of cohesin complex, is considered essential for meiotic chromosomal segregation (52). CHEK1 is an uncharacterized protein in pigs whose function may be associated with cell cycle arrest (53). In a word, all of these proteins contained in the above terms and pathways may play their respective roles in order to assure normal nuclear maturation of porcine oocytes.

The dynamic remodeling of cytoskeleton plays crucial roles in spindle assembly, chromosome segregation and organelle reorganization, ensuing proper nuclear and cytoplasmic maturation of mammalian oocytes (54). Based on the molecular function analysis, the terms related to microtubule regulation mainly enriched the over-expressed proteins and the majority of them belong to kinesin motor family of proteins (KIF11, KIF14, KIF15, KIF23, KIF4A, KIF18A, KIF20A, KIF20B, KIF2C, KIFC1). These proteins are essential for the functional mechanisms of macromolecule transport, microtubule dynamics, cell cycle progression and cytokinesis (55). It has been well known that dynamic changes in actin filaments are implicated in various events during oocyte meiotic maturation (56). In this study, we found that the enriched GO terms and KEGG pathways for under-expressed proteins contained actin-based functions. Among the proteins, myosin IB (MYO1B), myosin IE (MYO1E) and myosin VB (MYO5B) need specific attention, because they are numbers of the myosin family of motor proteins and also may have important roles in actin cytoskeleton remodeling during oocyte maturation.

It is established that the increase in progesterone induced by a luteinizing hormone is associated with meiotic resumption of porcine oocytes (57). As we expected, the KEGG pathways analysis showed that a total of 8 over-expressed proteins were involved in the “progesterone-mediated oocyte maturation” pathway, indicating their function in regulation of oocyte maturation. These proteins have been well elucidated as maternal proteins necessary for oogenesis. For instance, cyclin B1 (CCNB1) and proto-oncogene serine/threonine-protein kinase mos (MOS) are required for maturation promoting factor activation and mitogen-activated protein kinase cascade (58). The spindle checkpoint signaling depends on (BUB1) and serine/threonine-protein kinase PLK (PLK1) (59). Conversely, we found that the “cGMP-PKG signaling pathway” was significantly under-expressed in MII compared to GV oocytes, suggesting that this pathway was an important part of the regulatory mechanism related to meiotic maturation. Notable among these was the protein phosphatase 3 regulatory subunit B, alpha (PPP3R1) acting as the regulatory subunit of calcineurin B. It has been reported that calcineurin is present in porcine oocytes (60), and may modulate the meiotic maturation of mouse oocytes (61). Finally, another significantly enriched pathway was the Hippo signaling pathway, which has been speculated to be involved during oocyte maturation (62).

In this study, we also identified a series of DEPs related to epigenetic modifications including DNA methylation, histone acetylation and methylation. Among them, DNMT1, lymphoid specific helicase (HELLS), tudor domain containing 1 (TDRD1), tudor and TDRKH were enriched in the “DNA methylation or demethylation” term. It is worth mentioning that the expression of HELLS was elevated by 2.7-fold in MII oocytes, which as a DNA helicase is essential for genome-wide DNA methylation (63). In addition, GO enrichment analysis revealed the following enriched terms: “histone acetylation,” “histone H3 acetylation,” “H4 histone acetyltransferase complex,” and “histone acetyltransferase complex.” Both down-regulator of transcription 1 (DR1) and transcriptional adapter 3 (TADA3) were found to be involved in these terms, indicating that they might have a function during meiotic maturation in porcine oocytes through the modulation of histone deacetylation. However, the two proteins have hardly been studied previously in mammalian oocytes. The present study also showed that the retinoblastoma-binding family of proteins (RBBP4, RBBP5 and RBBP7) were linked to the formation of “histone methyltransferase complex” based on cellular component analysis. It has been reported that RBBP4 and RBBP7 can regulate histone deacetylation and chromosome segregation during mouse oocyte maturation (64, 65).

Conversely, our proteomic data were confirmed to be entirely reliable through PRM target validation for the selected 12 proteins. In the discussion above, WEE2, KIF20A, UBE2C, DNMT1, TDRKH and CPEB1 have been described to exert important roles in oocyte meiotic maturation. Moreover, GDF9, ANXA1 and PCNA are considered as oocyte-specific proteins and also identified in human oocytes through single-cell proteomics (15). It has been reported that CORO1C as an actin-binding protein is associated with cytogenesis in oocytes and embryos (66). According to a previous report (67), TPM4 might also be involved in the formation of microtubule structure in porcine oocyte maturation. Finally, our study found several cell surface glycoproteins with different expression including CD59, CD61, CD99 and CD276, suggesting that these proteins likely play important roles in porcine oocyte fertilization.

## Conclusion

In conclusion, we found 763 significantly altered proteins in GV and MII oocytes, suggesting that they might closely be related to oocyte maturation. Moreover, the functional classification and enrichment analysis revealed that these proteins were involved in multiple regulatory mechanisms of meiotic resumption and cytoplasmic maturation, such as spindle and chromosome configurations, cytoskeletal reconstruction, epigenetic modifications, energy metabolism, signal transduction and so on. All of these findings provide a deeper insight into the molecular characteristics of proteome in regulation of porcine oocyte maturation following IVM process.

## Data Availability Statement

The datasets presented in this study can be found in online repositories. The names of the repository/repositories and accession number(s) can be found at: http://www.proteomexchange.org/, PXD023107.

## Ethics Statement

Ethical review and approval was not required because live animals were not used in this study. Experiments carried out were according to institutional ethical guidelines and safety procedures.

## Author Contributions

BJ, DX, and GW conceived the experiments. BJ, DX, QS, QH, GQ, and GW conducted the experiments. BJ, DX, and GQ performed statistical analysis and figure generation. BJ, DX, and GW wrote the manuscript. GQ and GW reviewed the manuscript. All authors have read and agreed to the published version of the manuscript.

## Funding

This work was supported by National Natural Science Foundation of China (Nos. 31660661, 32160793, and 31960663), Yunnan Applied Basic Research Projects (Nos. 202001AS070001 and 202101AT070213), and Yunnan Young Academic Leader Program (No. 202005AC160004).

## Conflict of Interest

The authors declare that the research was conducted in the absence of any commercial or financial relationships that could be construed as a potential conflict of interest.

## Publisher's Note

All claims expressed in this article are solely those of the authors and do not necessarily represent those of their affiliated organizations, or those of the publisher, the editors and the reviewers. Any product that may be evaluated in this article, or claim that may be made by its manufacturer, is not guaranteed or endorsed by the publisher.

## Supplementary Material

Supplementary Figure S1 A principal component analysis of all quantified proteins.

Supplementary Figure S2 The fragment ion peak area distribution of identified peptides.

Supplementary Table S1 Annotation of quantified proteins.

Supplementary Table S2 Differentially expressed proteins.

Supplementary Table S3 GO classification analysis.

Supplementary Table S4 COG/KOG classification analysis.

Supplementary Table S5 GO enrichment analysis.

Supplementary Table S6 KEGG pathway enrichment analysis.

Supplementary Table S7 Cluster analysis of enriched GO terms.
