# Supplementary figures and images for: Proteomic Exploration of Porcine Oocytes During Meiotic Maturation in vitro Using an Accurate TMT-Based Quantitative Approach
Source: Front Vet Sci. 2022 Feb 7;8:792869. doi: 10.3389/fvets.2021.792869 (PMC8859466; doi:10.3389/fvets.2021.792869)

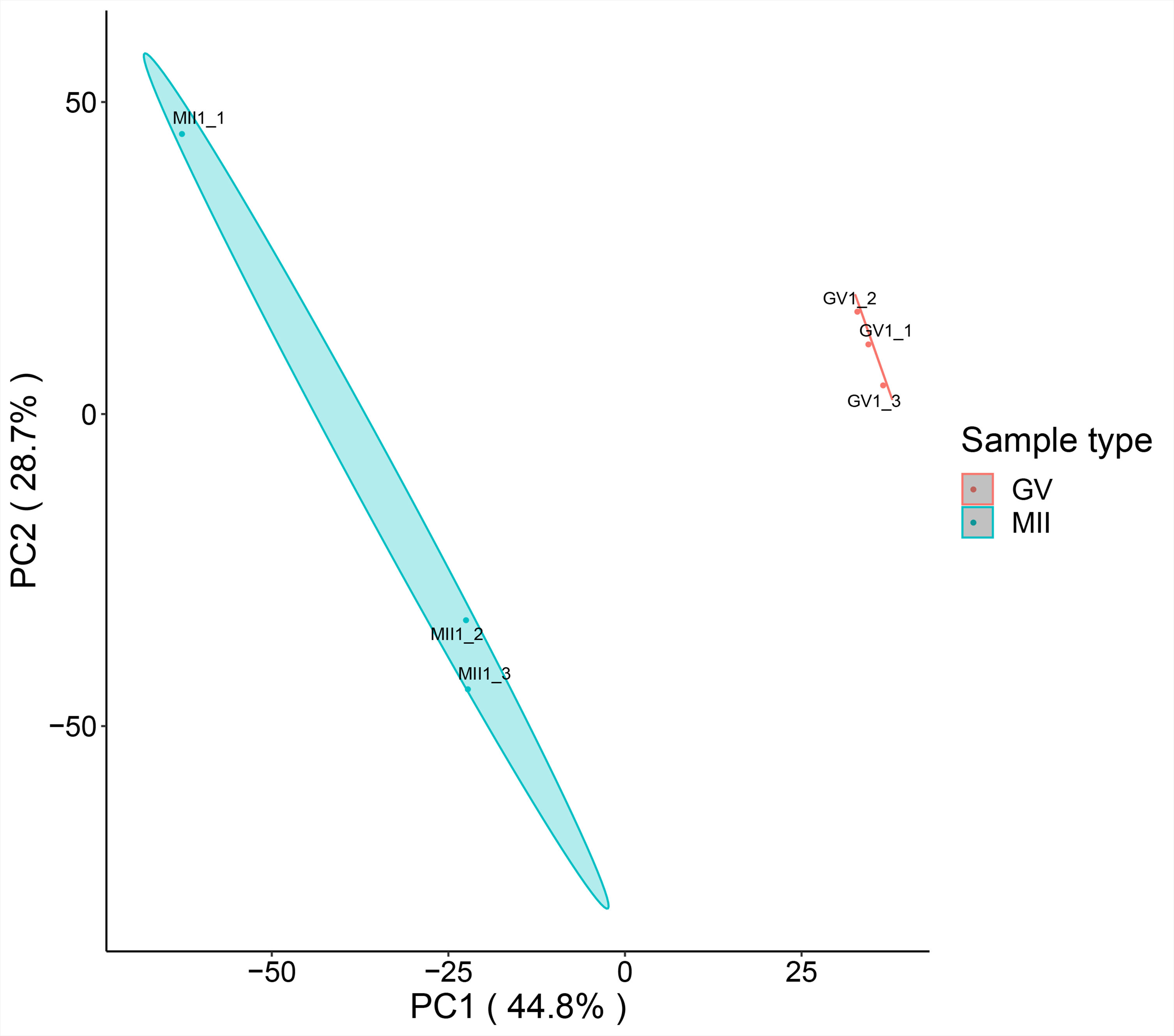

Supplement: Supplementary Figure S1 — A principal component analysis of all quantified proteins. [file Image_1.tif]
