# Supplementary material for: Proteomic Exploration of Porcine Oocytes During Meiotic Maturation in vitro Using an Accurate TMT-Based Quantitative Approach
Source: Front Vet Sci. 2022 Feb 7;8:792869. doi: 10.3389/fvets.2021.792869 (PMC8859466; doi:10.3389/fvets.2021.792869)
Supplement: Supplementary Figure S2 — The fragment ion peak area distribution of identified peptides. [file Image_2.pdf]

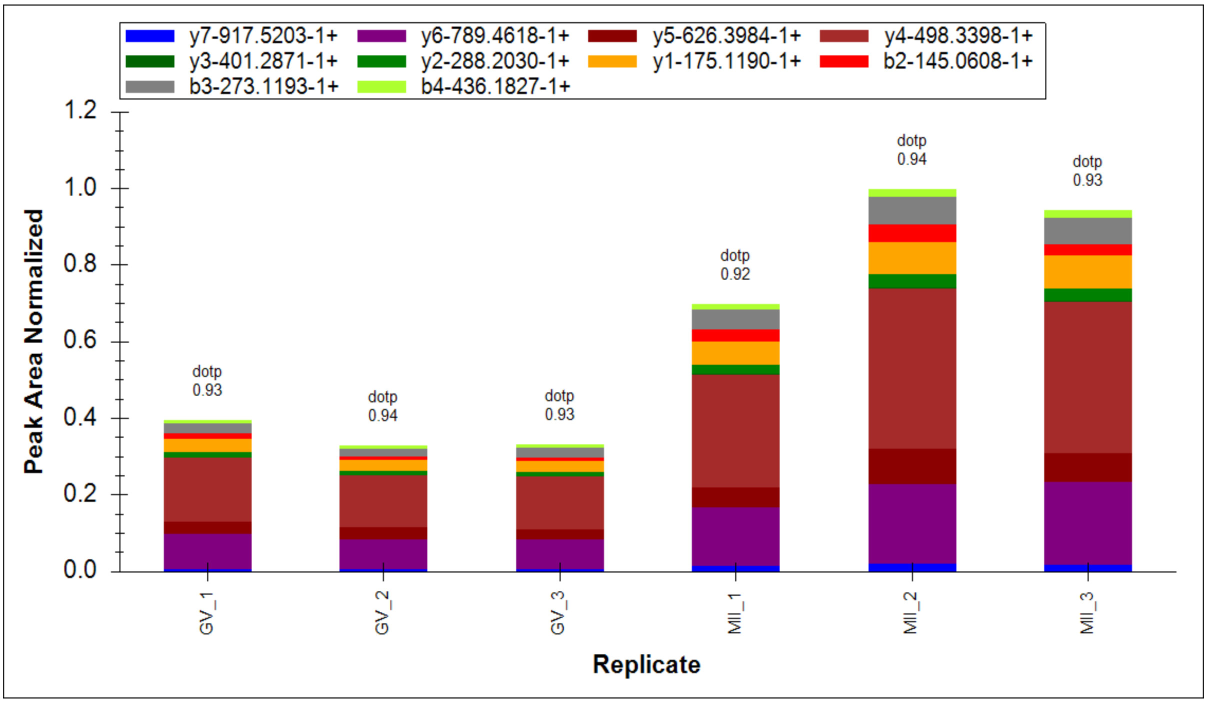

GSQYQPILR peptide of A0A287A8G0 protein

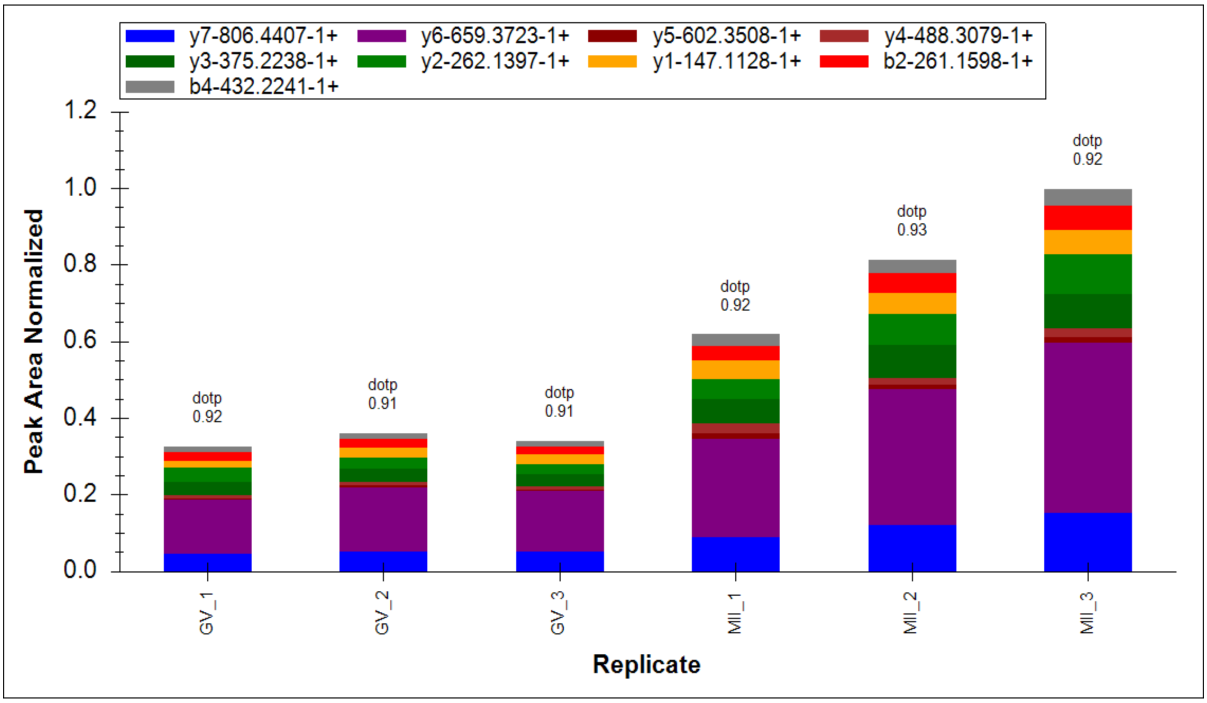

LFGNILDK peptide of A0A287A8G0 protein

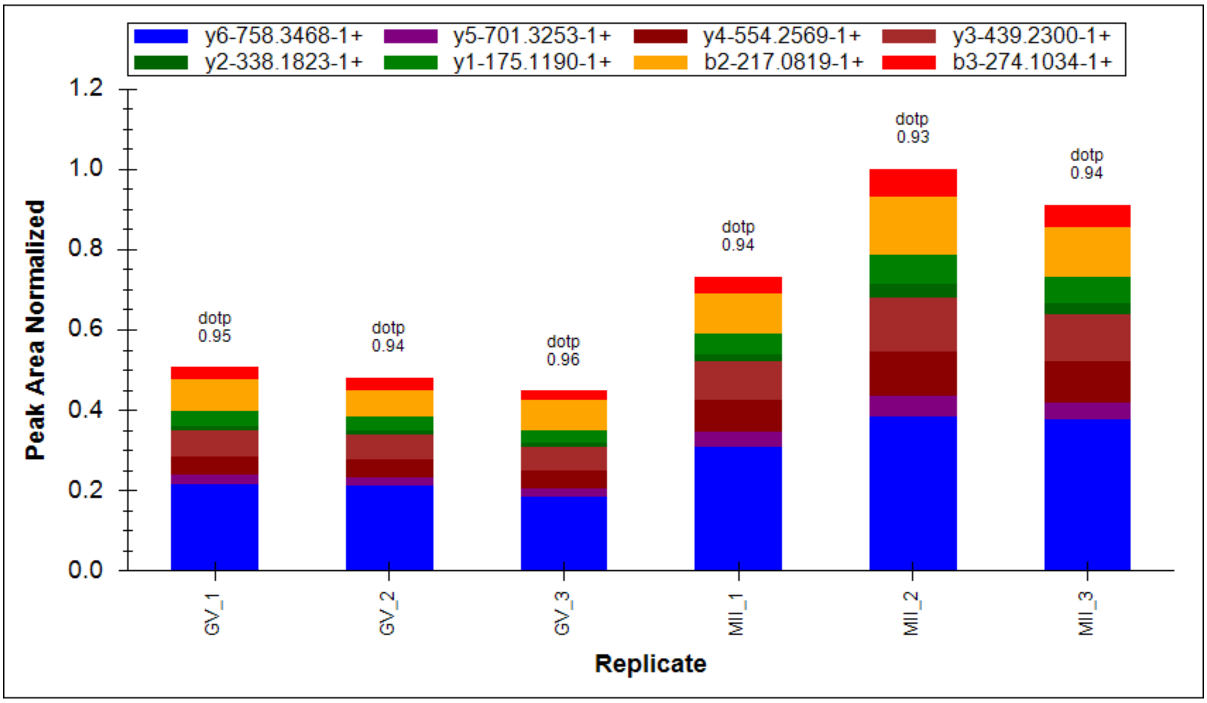

SEGFDTYR peptide of A0A287ABG2 protein

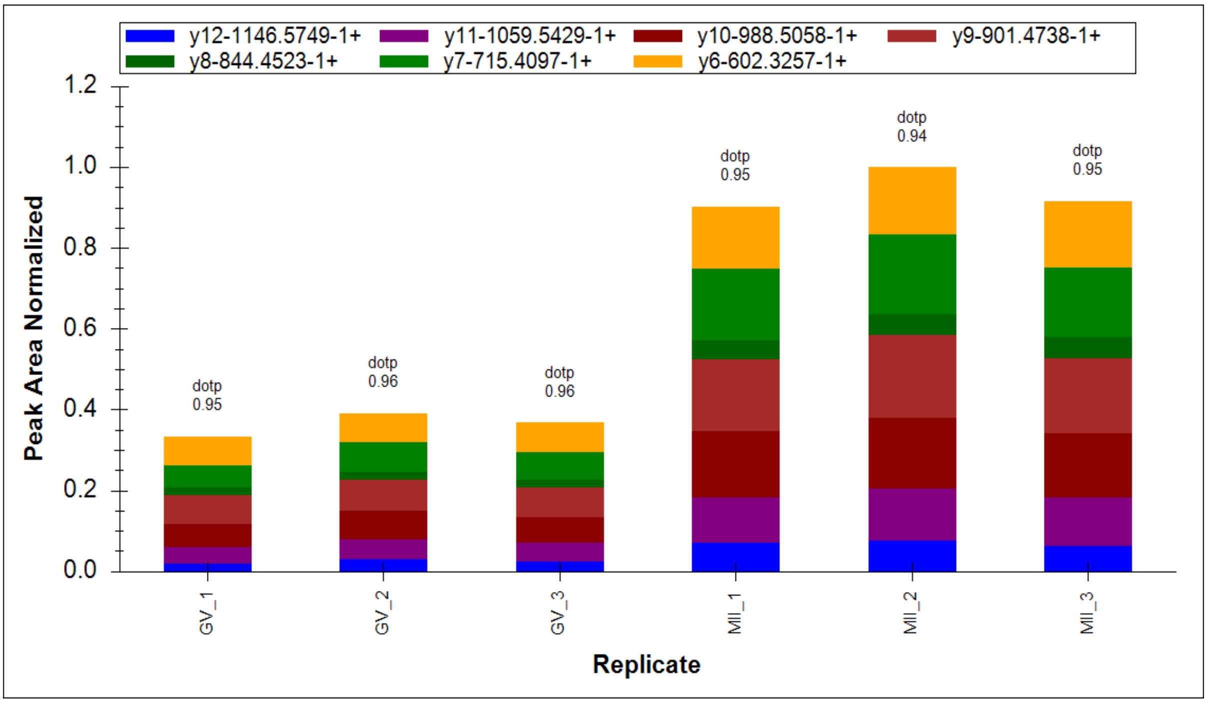

FSASGELGNGNIK peptide of A0A287ABG2 protein

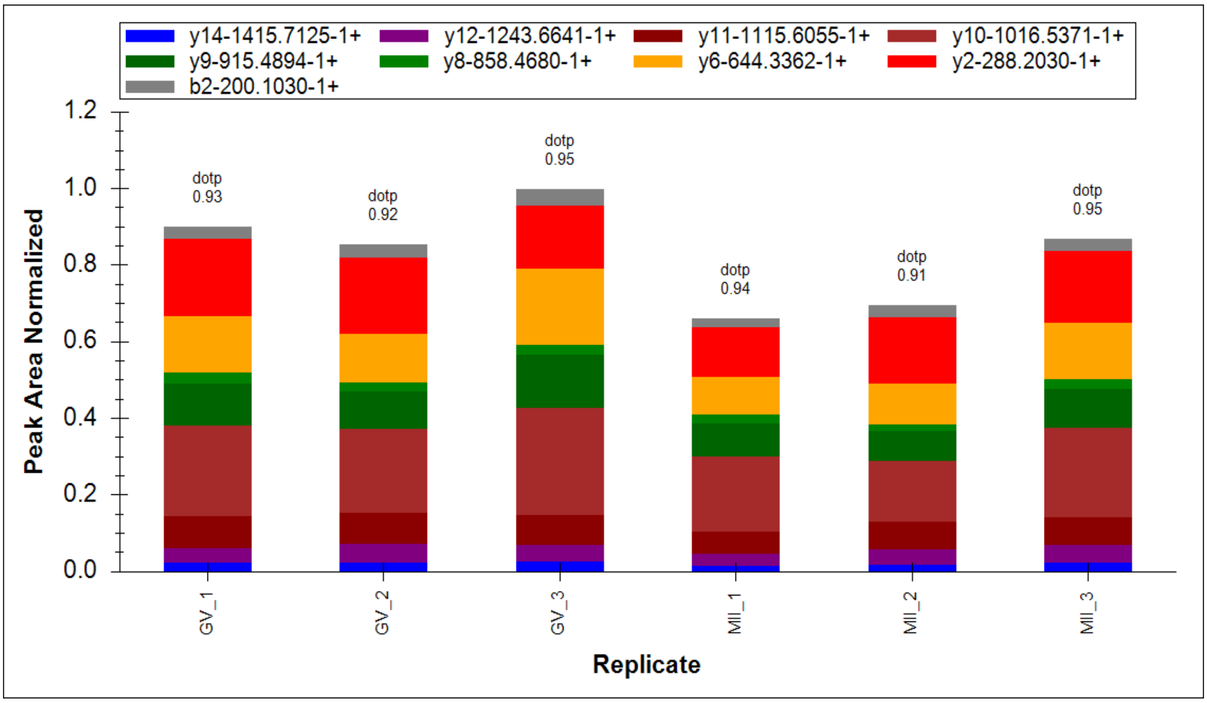

QAPGDQVTGTLPSGDLR peptide of Q6GUA6 protein

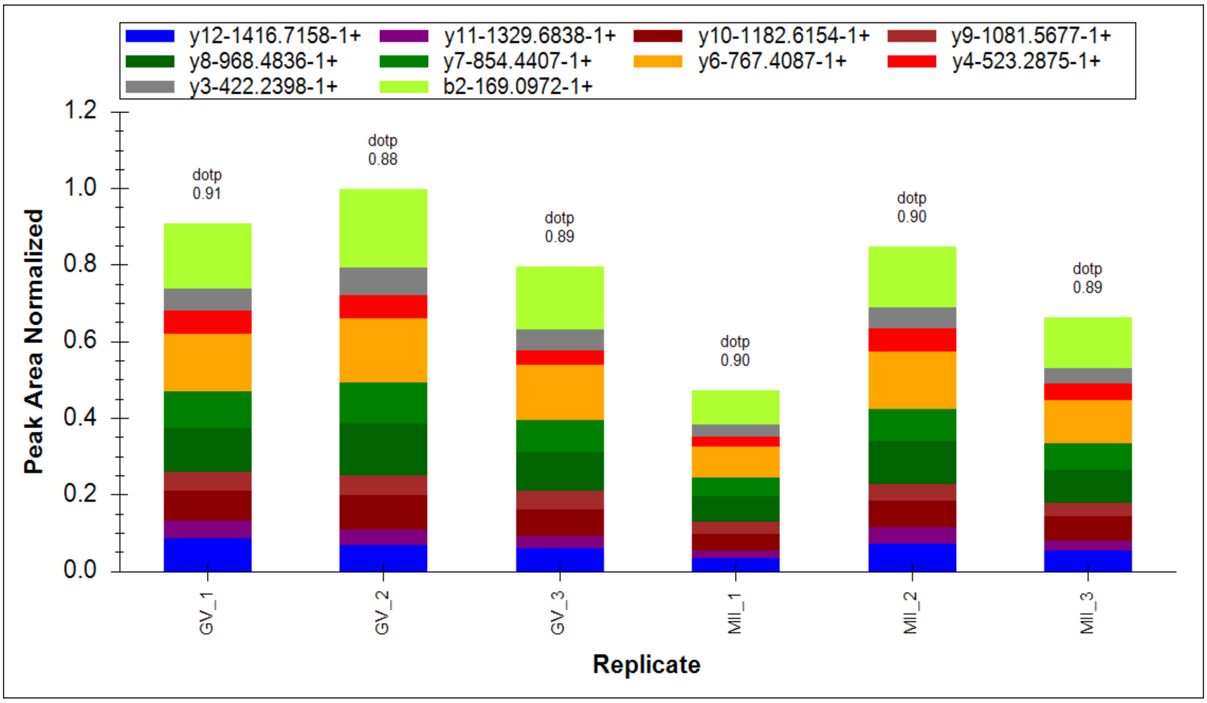

APYSFTLNSPFTFQK peptide of Q6GUA6 protein

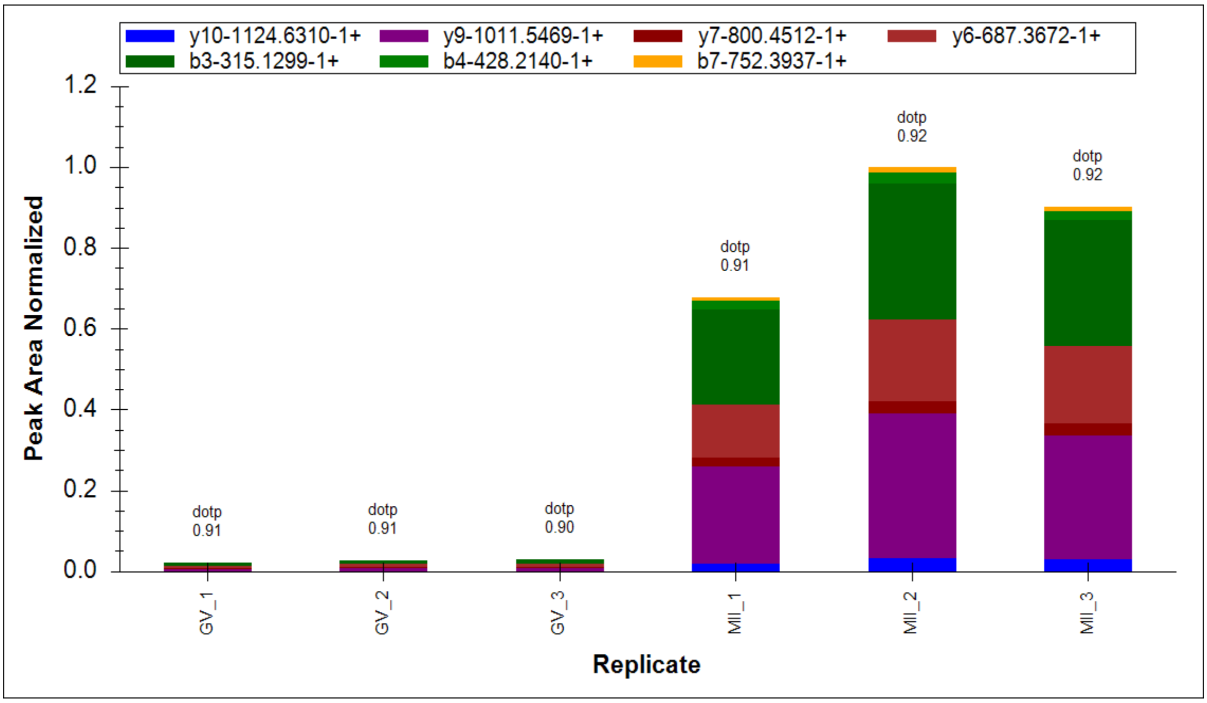

EGQLPNIPQDLSK peptide of A4PES0 protein

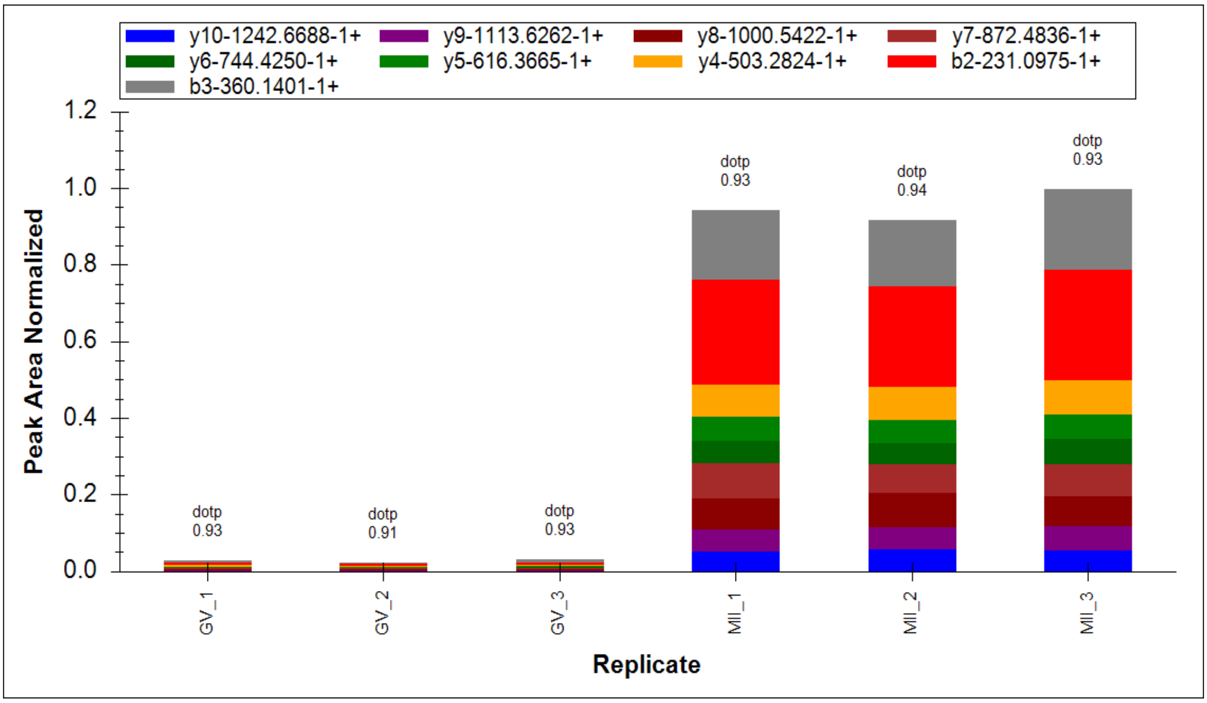

TEELQQQLNLEK peptide of A4PES0 protein

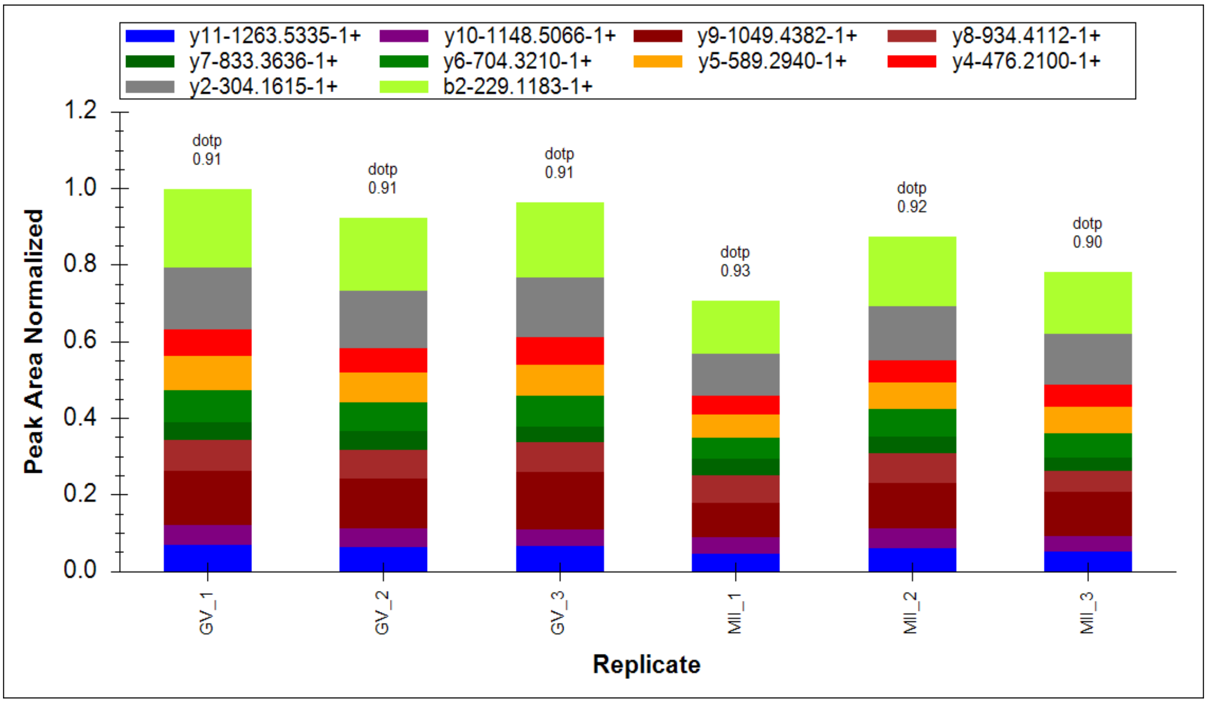

IDVDTEDIGDER peptide of I3LP40 protein

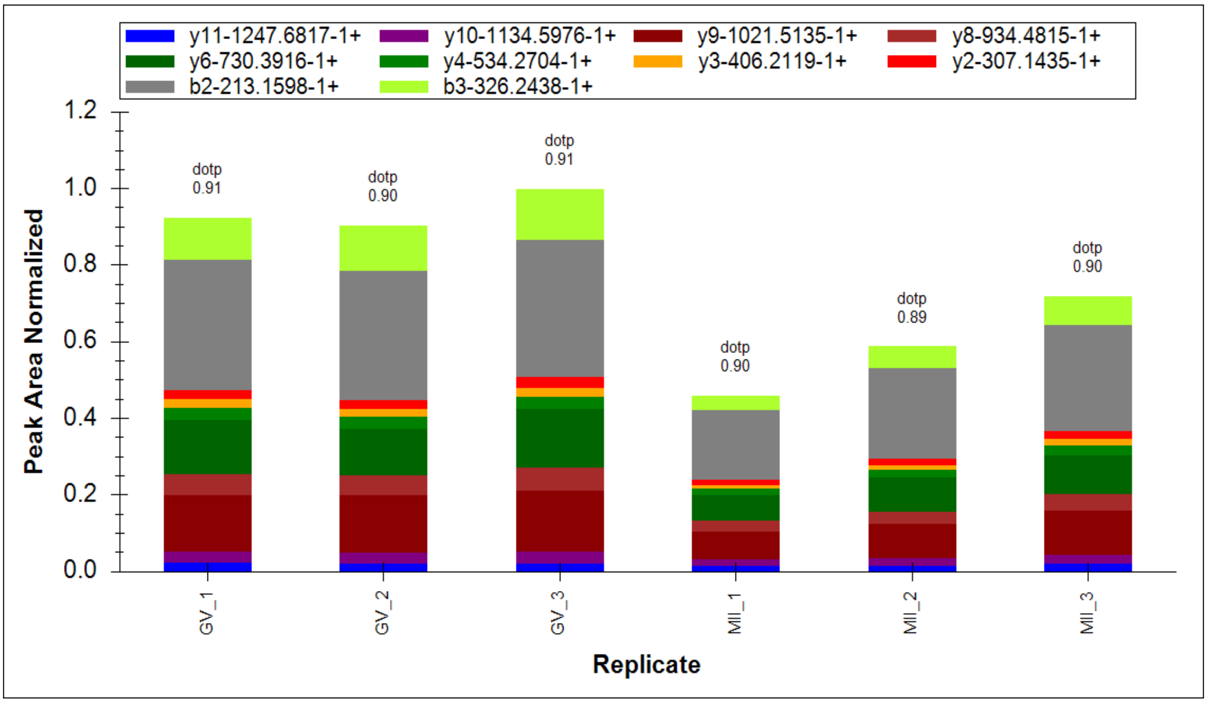

VLLISGFPVQVCK peptide of I3LP40 protein

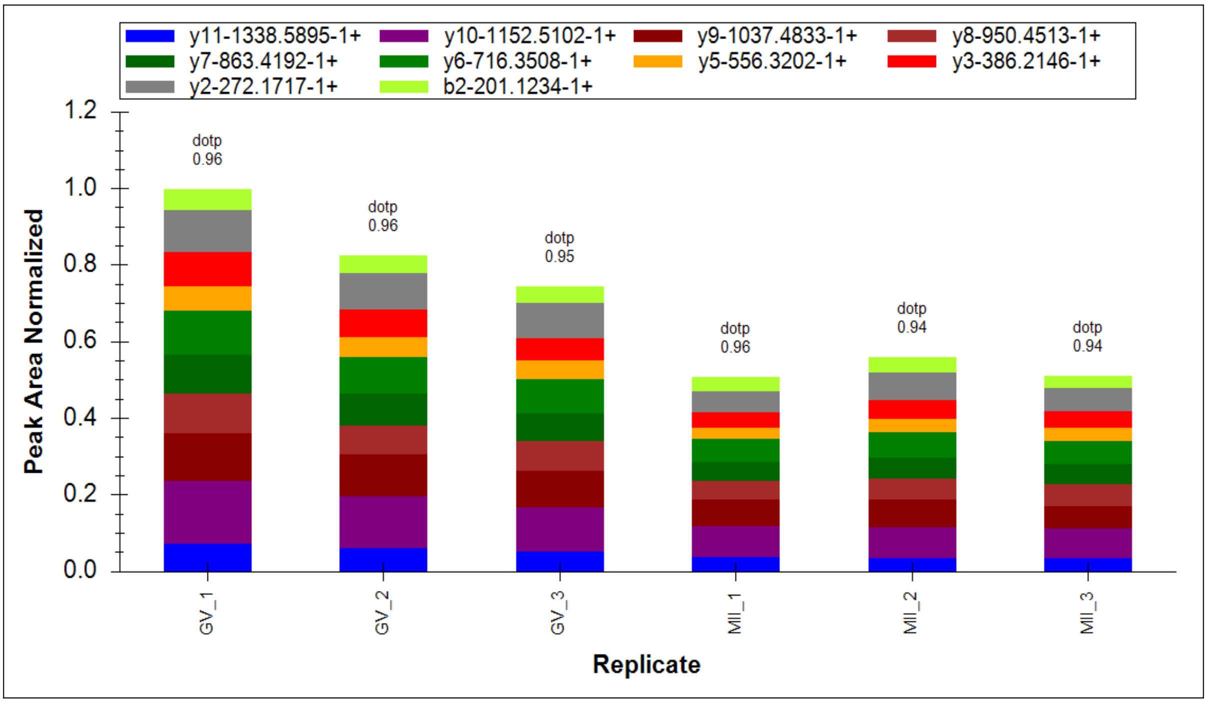

VTWDSSFCAVNPR peptide of F1RGA9 protein

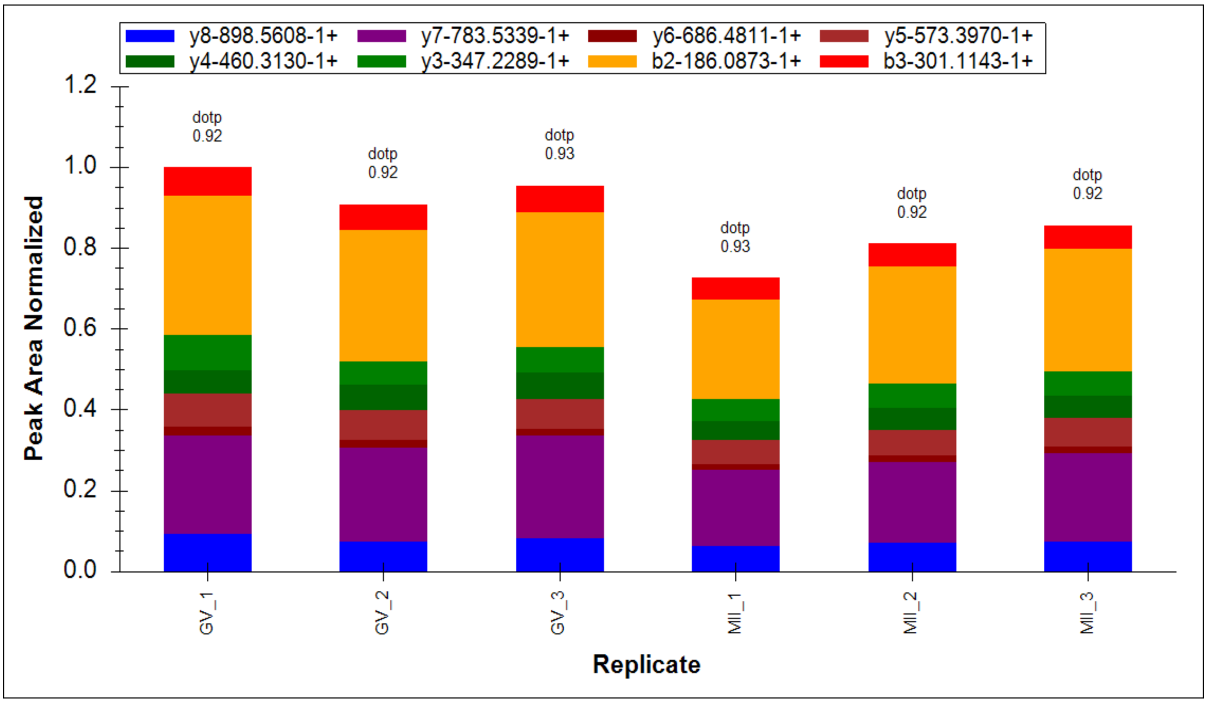

NADPILISLK peptide of F1RGA9 protein

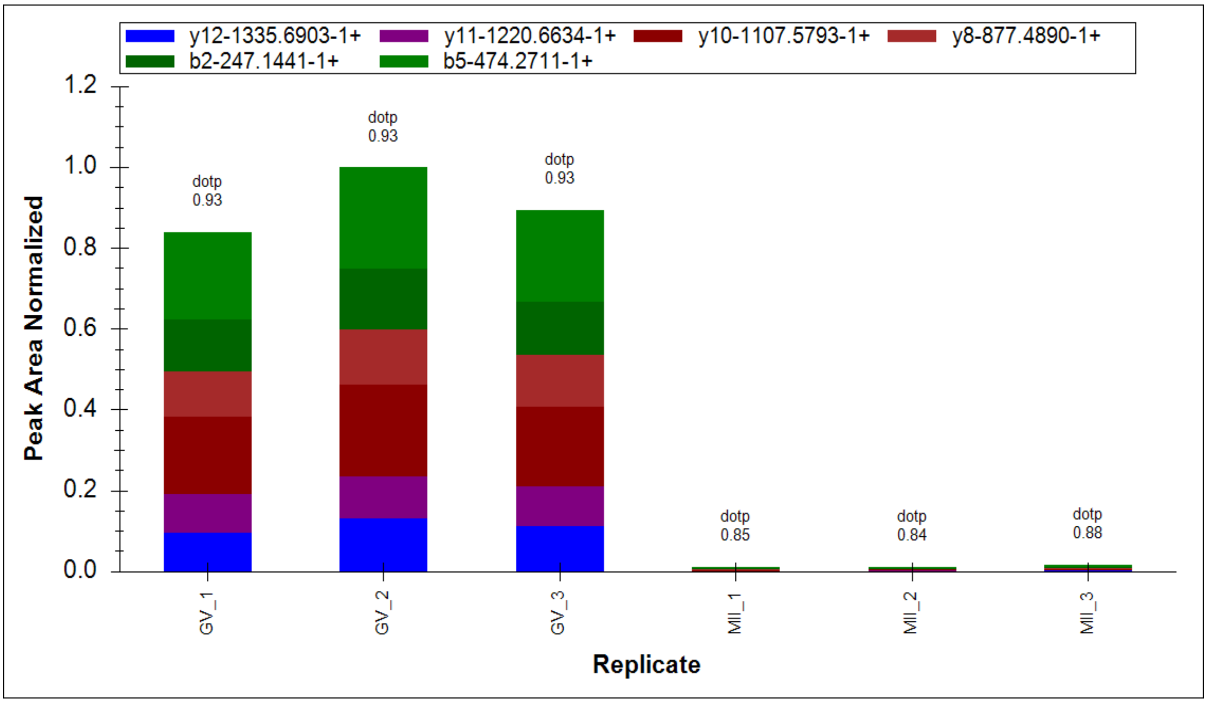

VFLGGVPWDITEAGLVNTFR peptide (+2) of F1RI92 protein

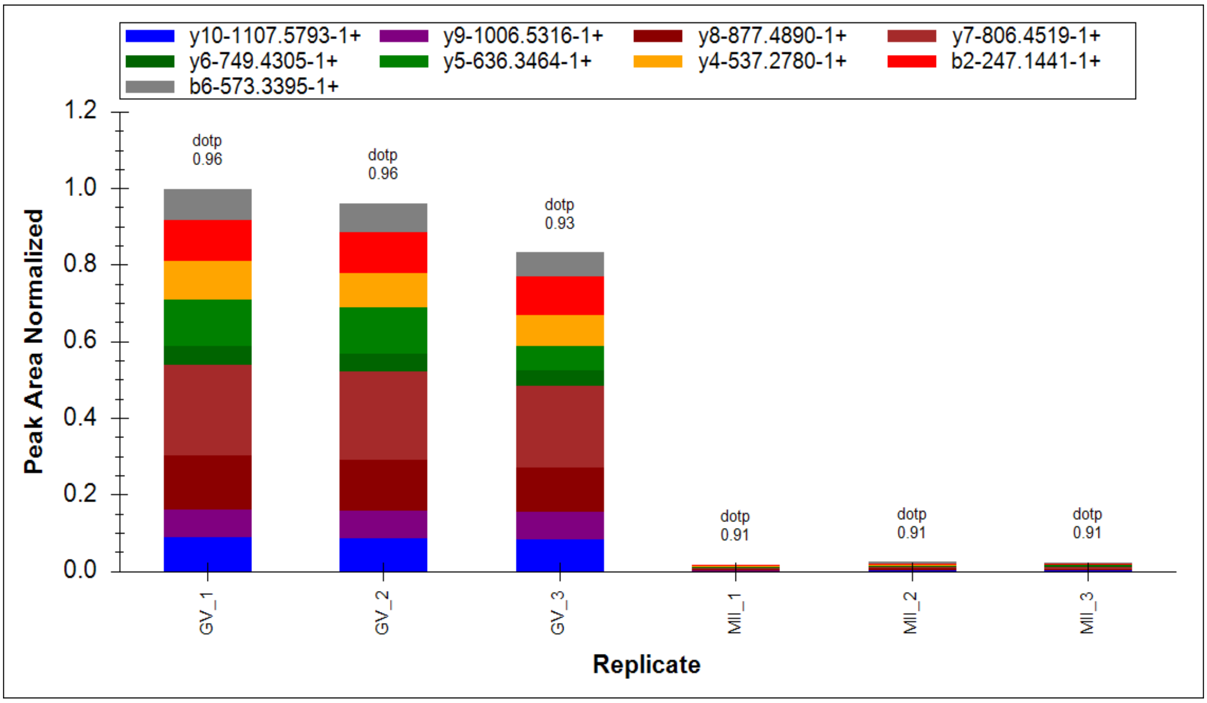

VFLGGVPWDITEAGLVNTFR peptide (+3) of F1RI92 protein

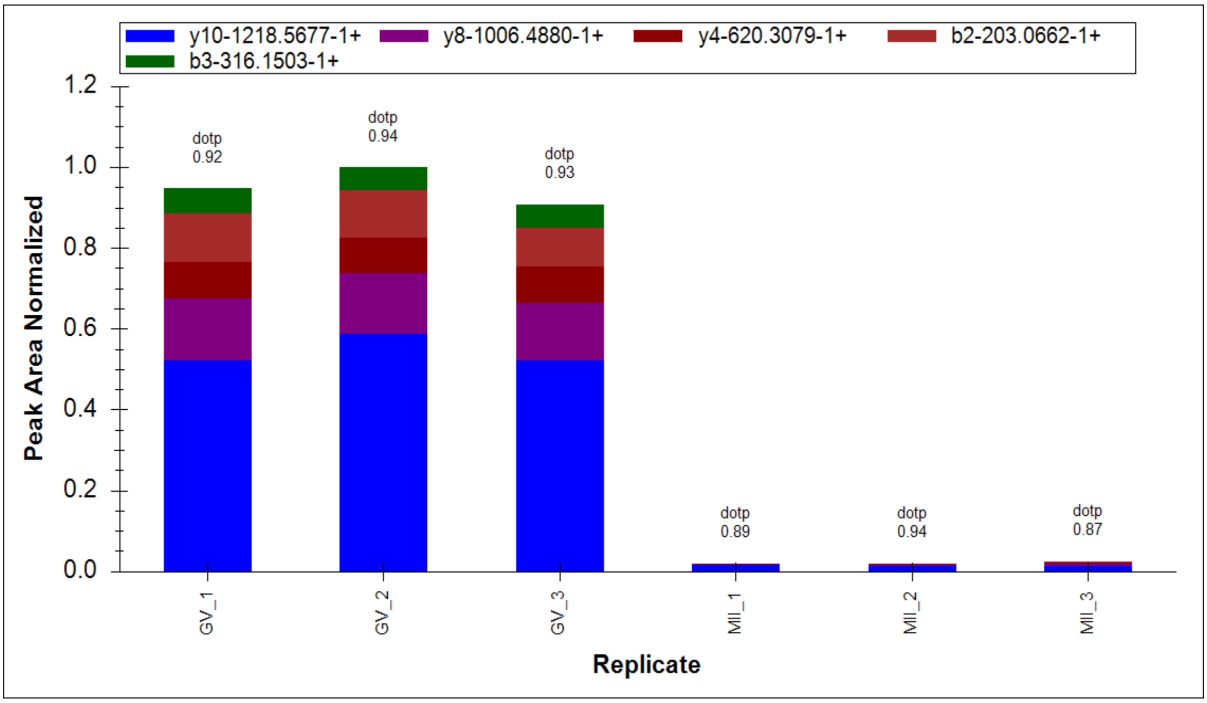

DSLNPDGLSEYYFK peptide of F1RI92 protein

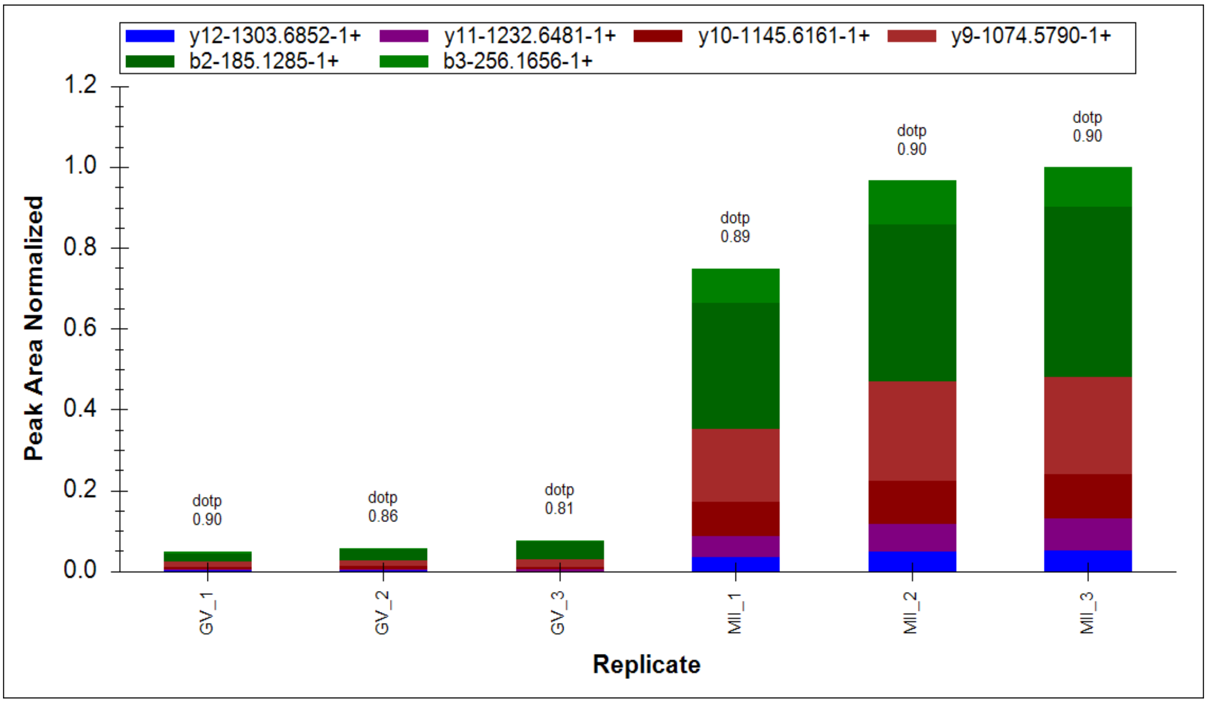

LAASASTQQLQEIK peptide of F1RH90 protein

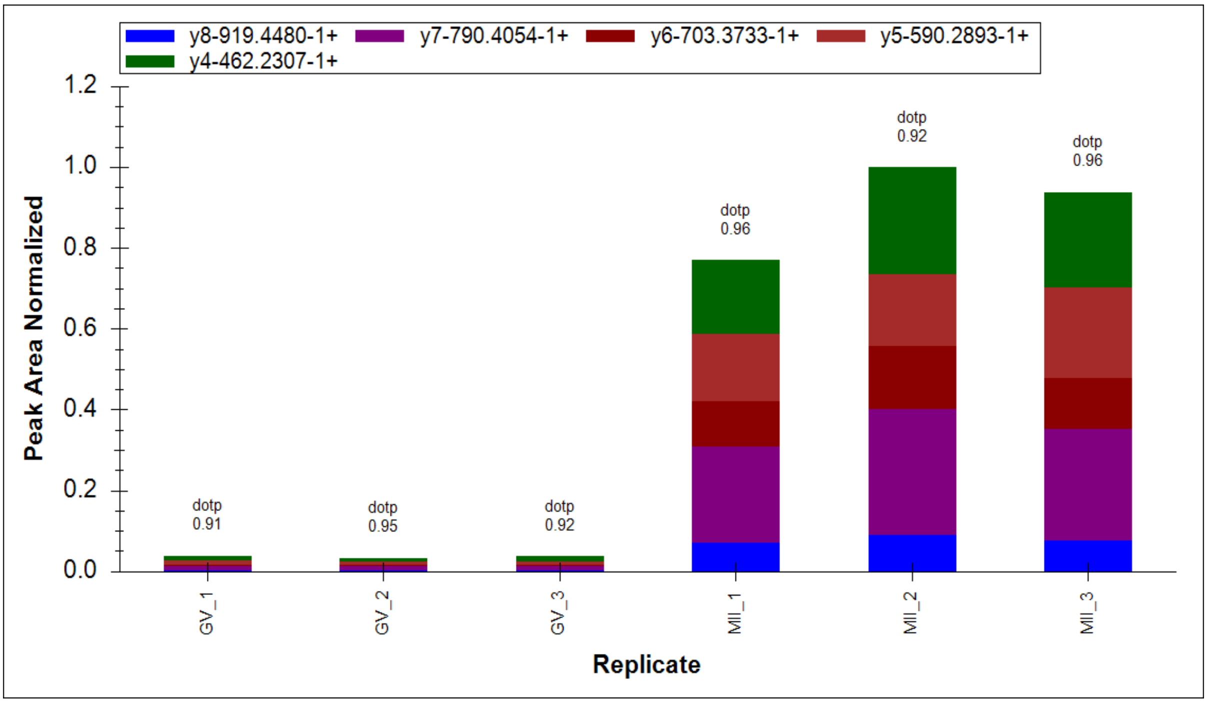

LGESLQSAER peptide of F1RH90 protein

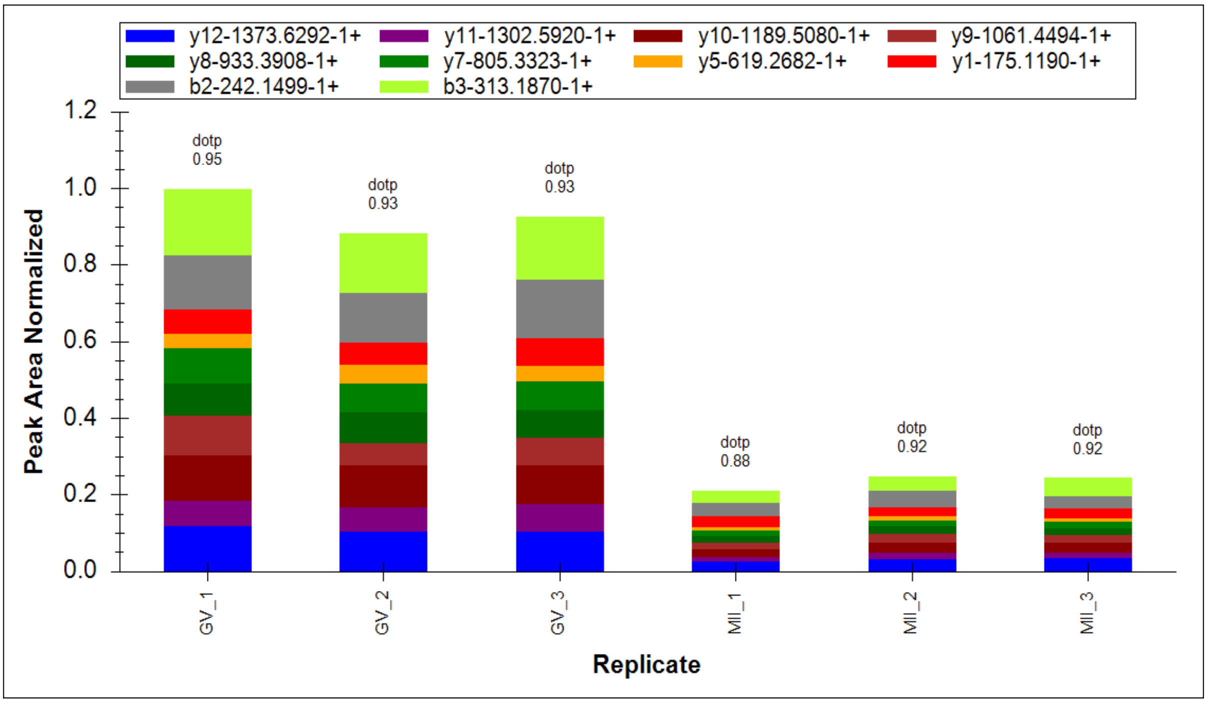

IQALQQQADEAEDR peptide of P67937 protein

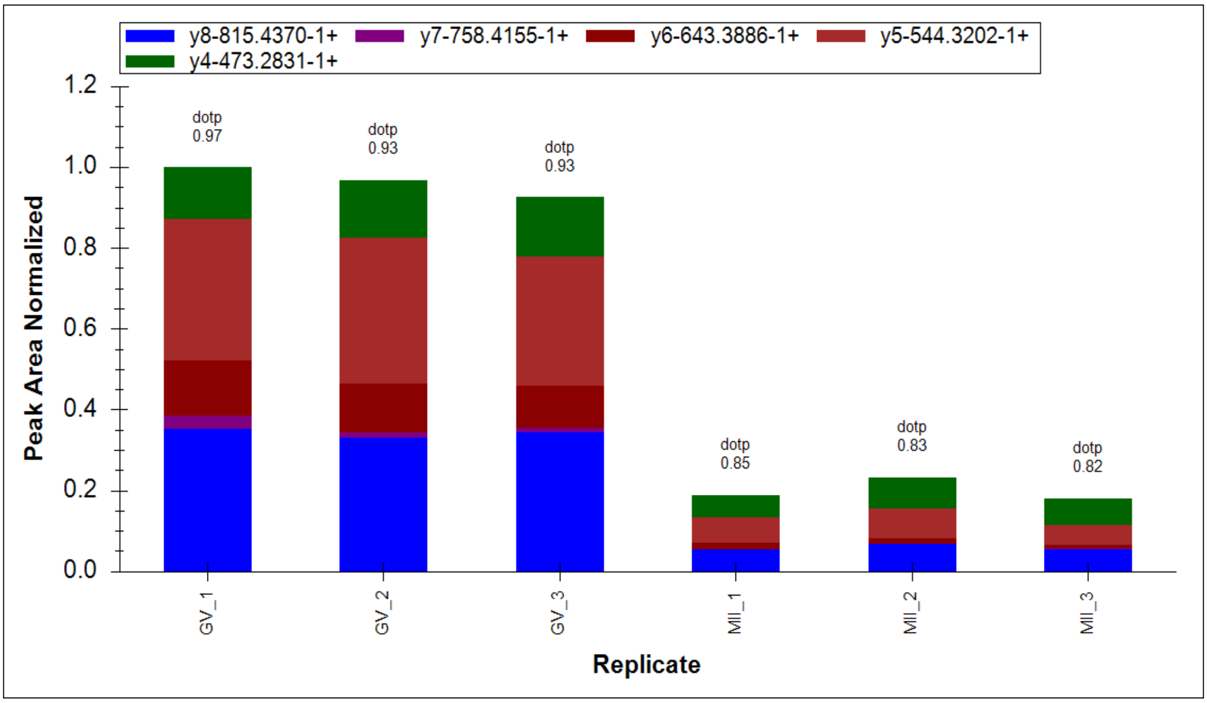

AEGDVAALNR peptide of P67937 protein

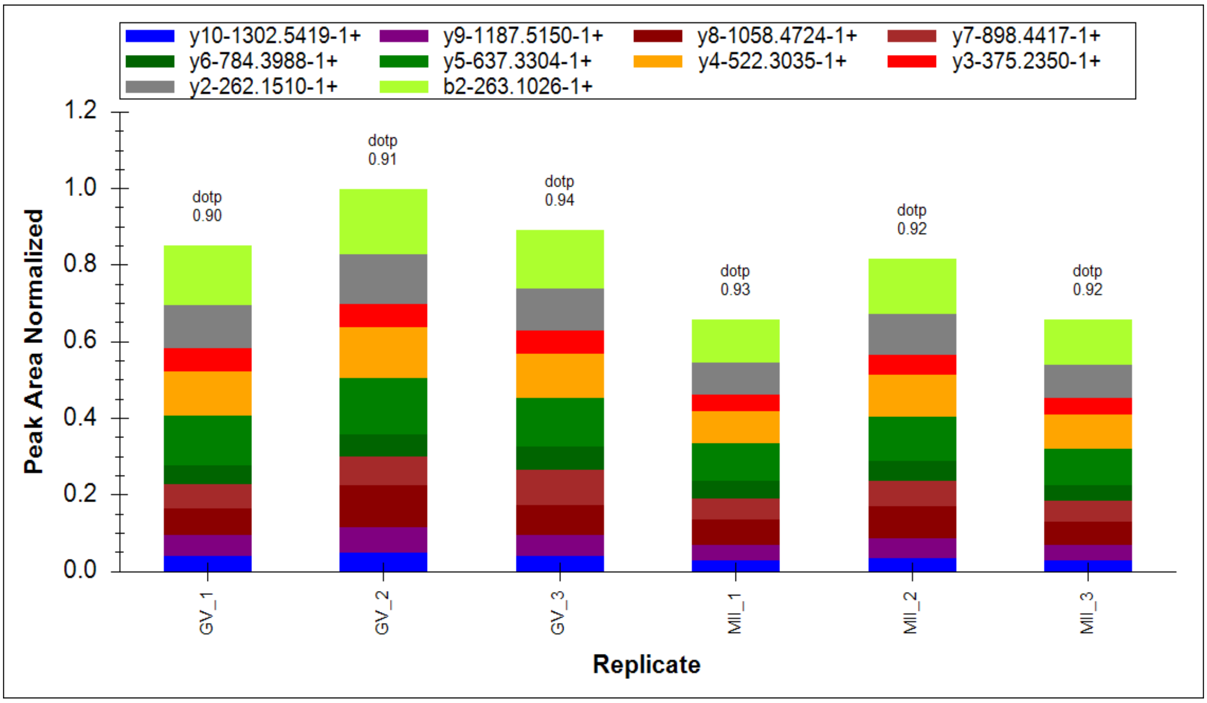

FDECNFDFISR peptide of O62680 protein

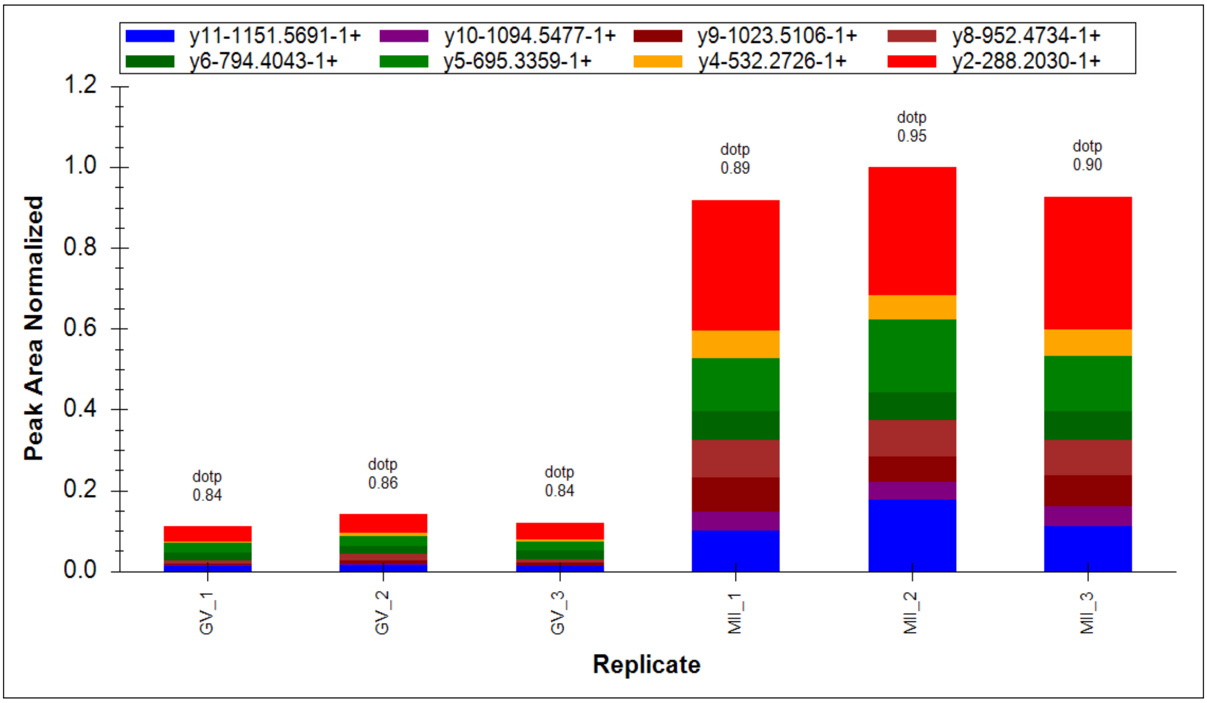

WVGTIHGAAGTVYEDLR peptide of F1SC78 protein

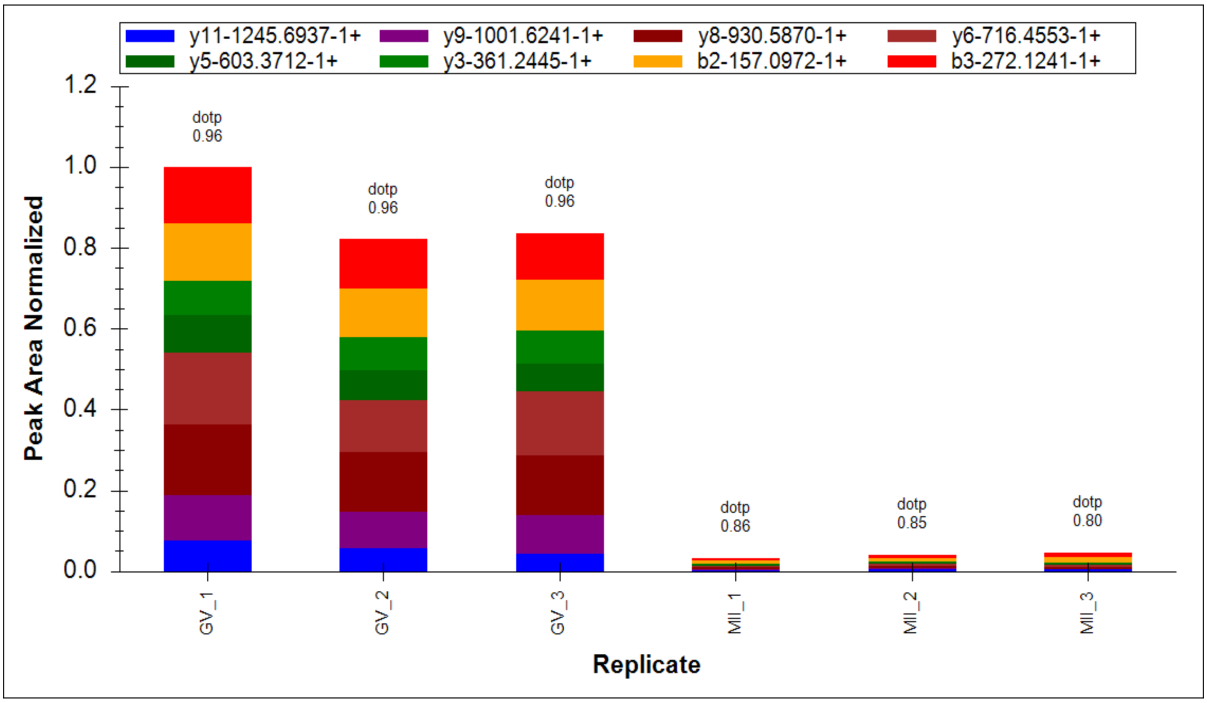

GVDEATIIEILTK peptide of F1SJB5 protein

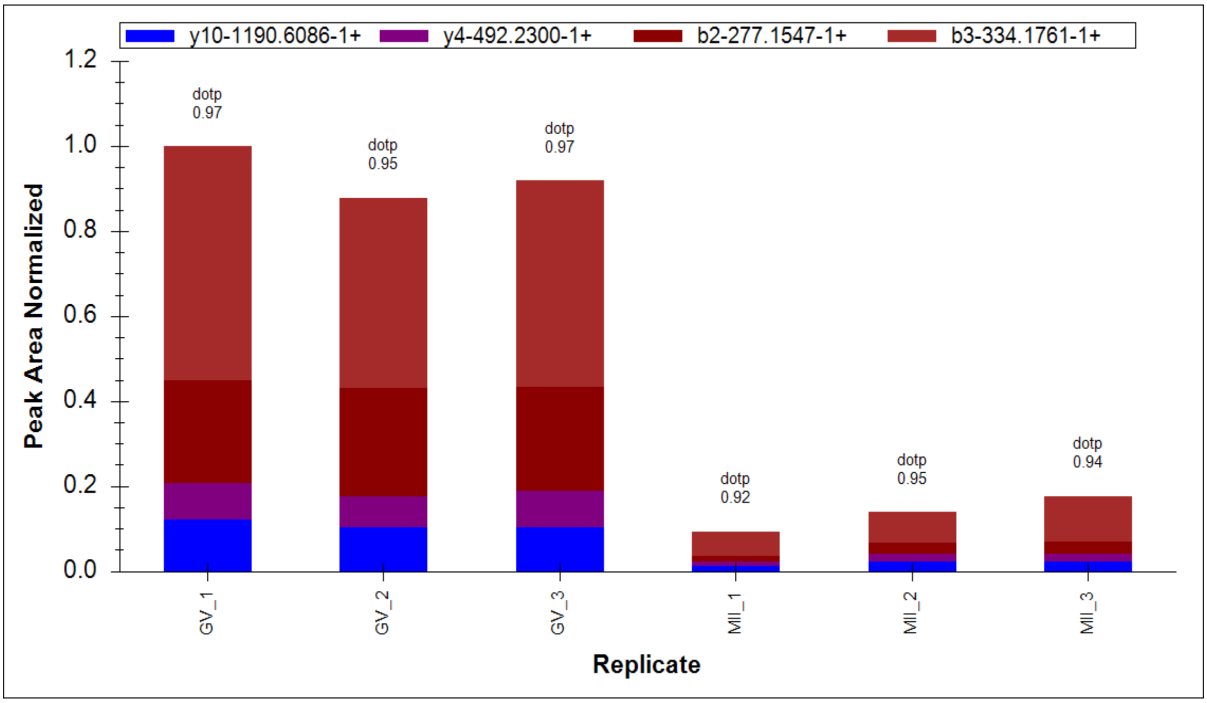

LYGISLCQAILDETK peptide of F1SJB5 protein
